# Supplementary figures and images for: HSC70 coordinates COP9 signalosome and SCF ubiquitin ligase activity to enable a prompt stress response
Source: EMBO Rep. 2025 Feb 6;26(5):1344–66. doi: 10.1038/s44319-025-00376-x (PMC11894141; doi:10.1038/s44319-025-00376-x)

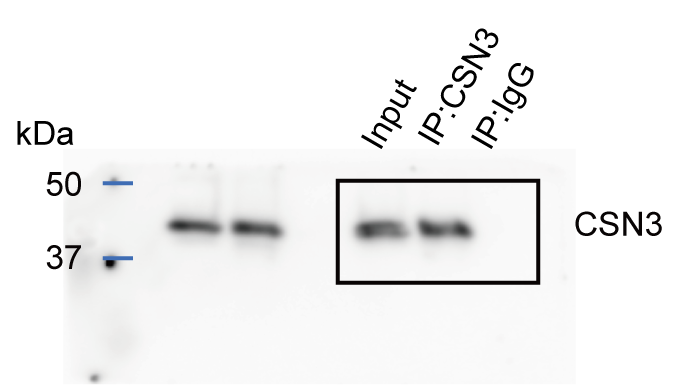

Supplement: Supplementary file 2 — Source data Fig. 1 [file 44319_2025_376_MOESM2_ESM.zip › Fig.1/Fig.1B/CSN3-IP IB CSN3/Fig.1B CSN3-IP (IB CSN3).tif]

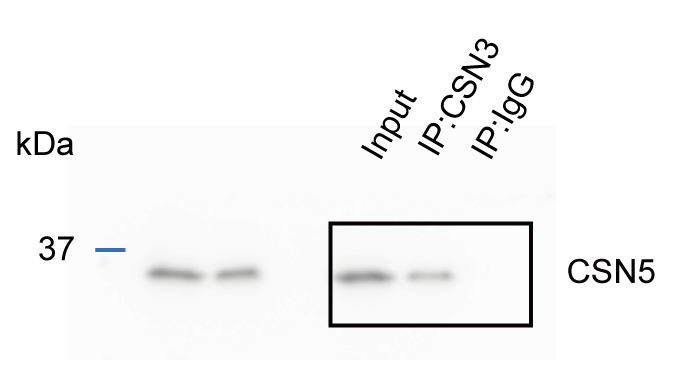

Supplement: Supplementary file 2 — Source data Fig. 1 [file 44319_2025_376_MOESM2_ESM.zip › Fig.1/Fig.1B/CSN3-IP IB CSN5/Fig.1B CSN3-IP (IB CSN5).tif]

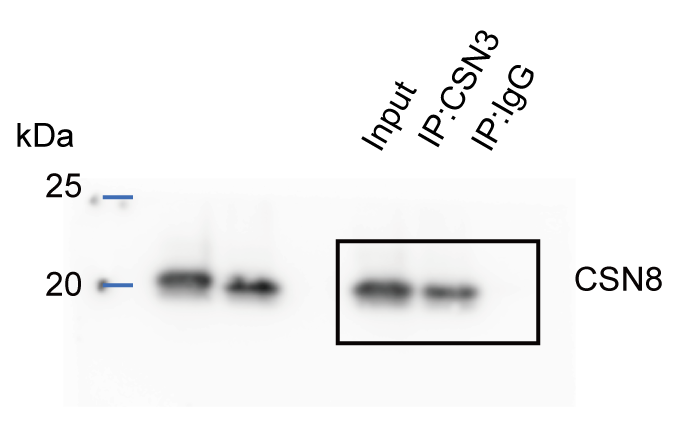

Supplement: Supplementary file 2 — Source data Fig. 1 [file 44319_2025_376_MOESM2_ESM.zip › Fig.1/Fig.1B/CSN3-IP IB CSN8/Fig.1B CSN3-IP (IB CSN8).tif]

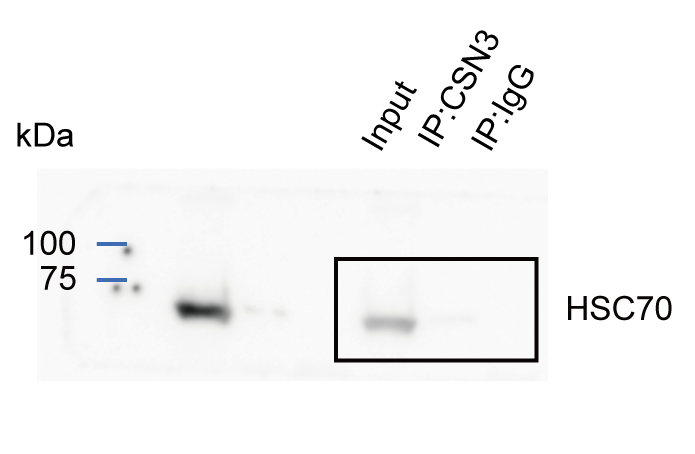

Supplement: Supplementary file 2 — Source data Fig. 1 [file 44319_2025_376_MOESM2_ESM.zip › Fig.1/Fig.1B/CSN3-IP IB HSC70/Fig.1B CSN3-IP (IB HSC70).tif]

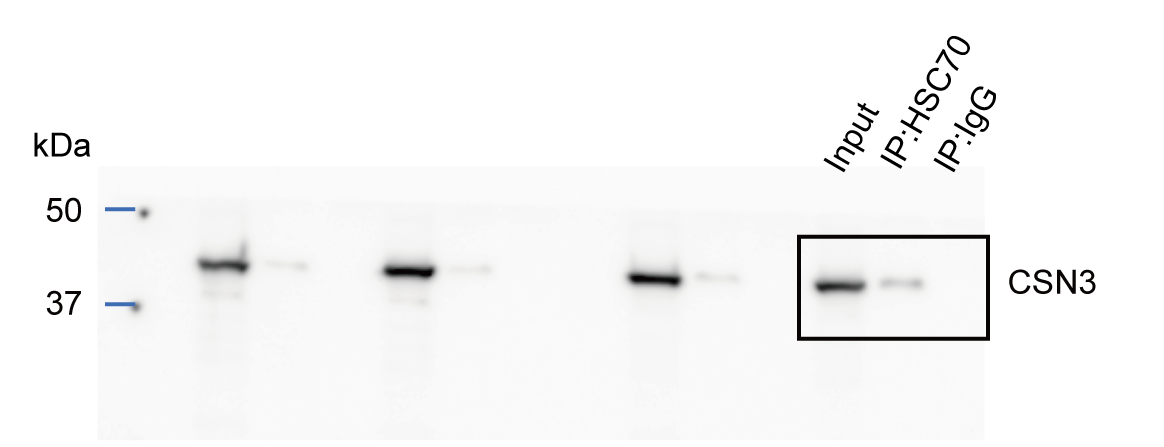

Supplement: Supplementary file 2 — Source data Fig. 1 [file 44319_2025_376_MOESM2_ESM.zip › Fig.1/Fig.1B/HSC70-IP IB CSN3/Fig.1B HSC70-IP (IB CSN3).tif]

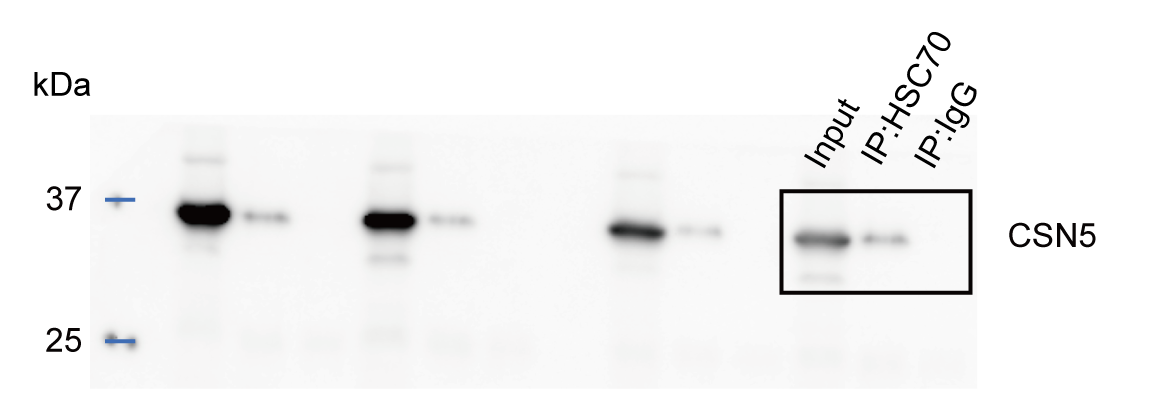

Supplement: Supplementary file 2 — Source data Fig. 1 [file 44319_2025_376_MOESM2_ESM.zip › Fig.1/Fig.1B/HSC70-IP IB CSN5/Fig.1B HSC70-IP (IB CSN5).tif]

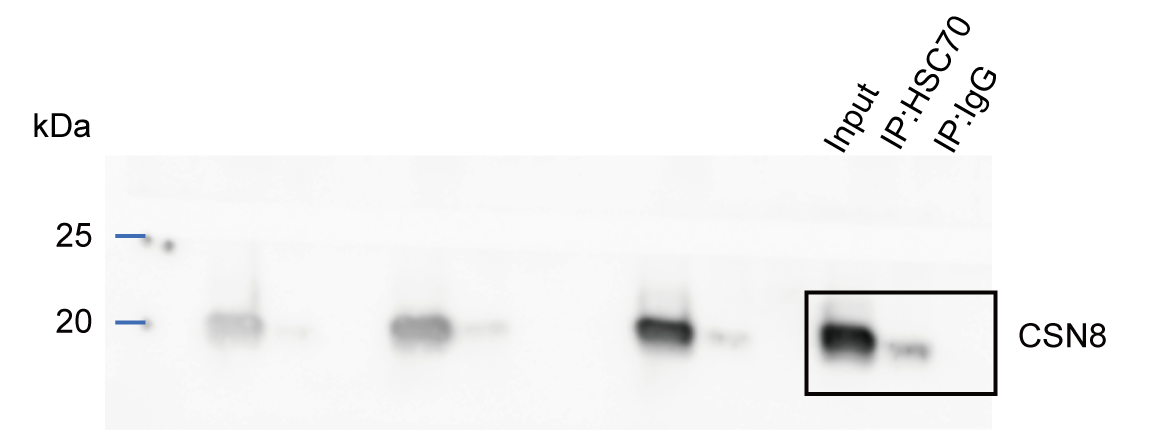

Supplement: Supplementary file 2 — Source data Fig. 1 [file 44319_2025_376_MOESM2_ESM.zip › Fig.1/Fig.1B/HSC70-IP IB CSN8/Fig.1B HSC70-IP (IB CSN8).tif]

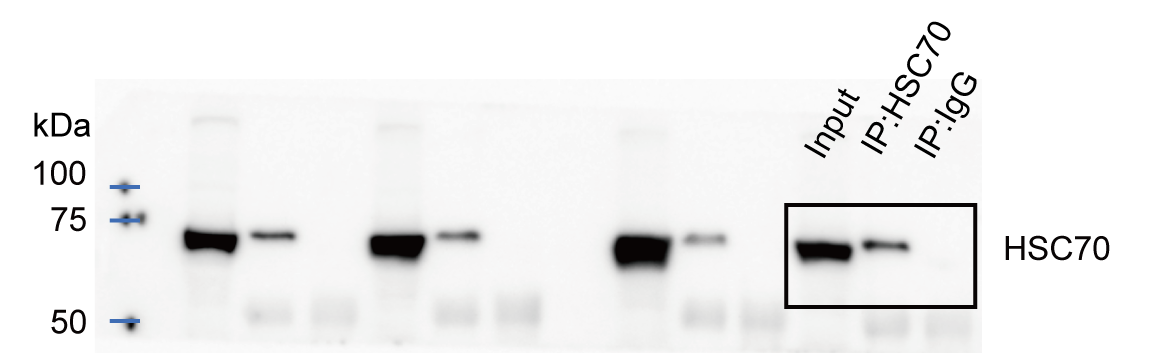

Supplement: Supplementary file 2 — Source data Fig. 1 [file 44319_2025_376_MOESM2_ESM.zip › Fig.1/Fig.1B/HSC70-IP IB HSC70/Fig.1B HSC70-IP (IB HSC70).tif]

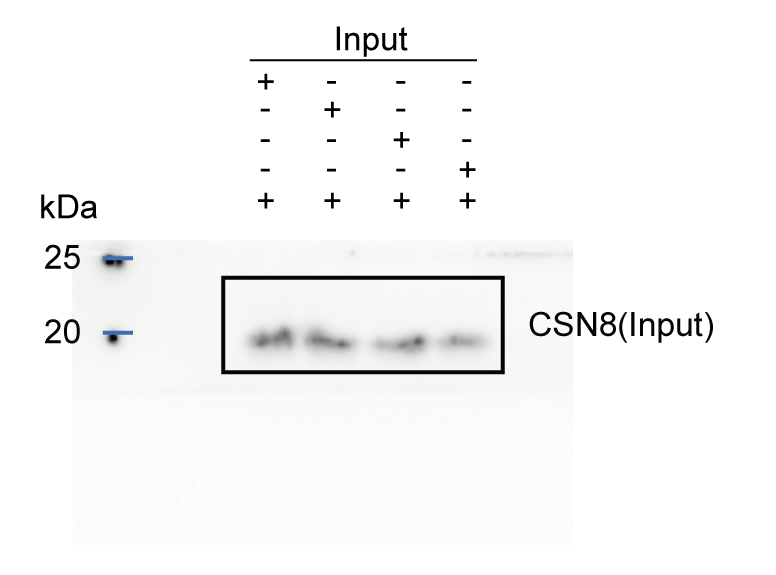

Supplement: Supplementary file 2 — Source data Fig. 1 [file 44319_2025_376_MOESM2_ESM.zip › Fig.1/Fig.1D/CSN8 Input/Fig.1D CSN8 (Input).tif]

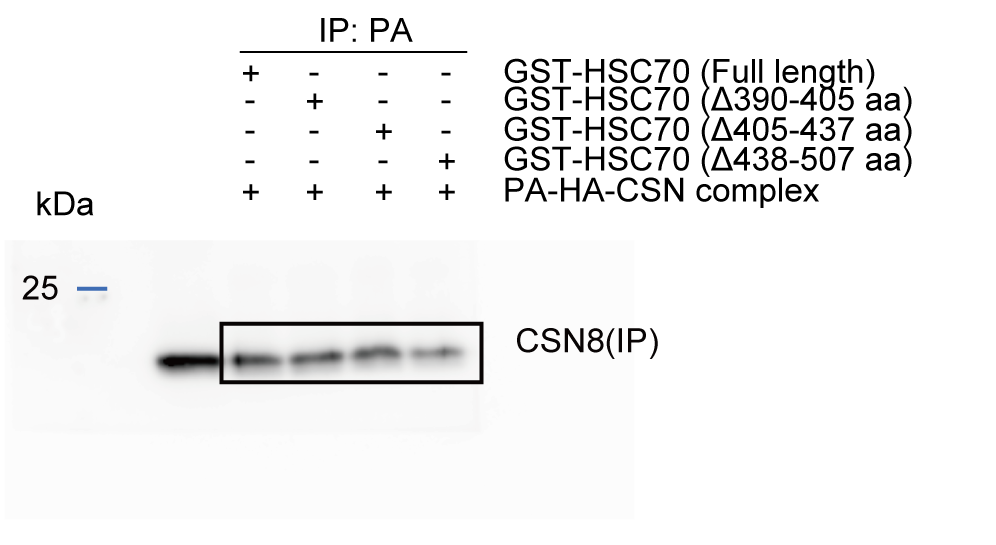

Supplement: Supplementary file 2 — Source data Fig. 1 [file 44319_2025_376_MOESM2_ESM.zip › Fig.1/Fig.1D/CSN8 IP/Fig.1D CSN8 (PA-IP).tif]

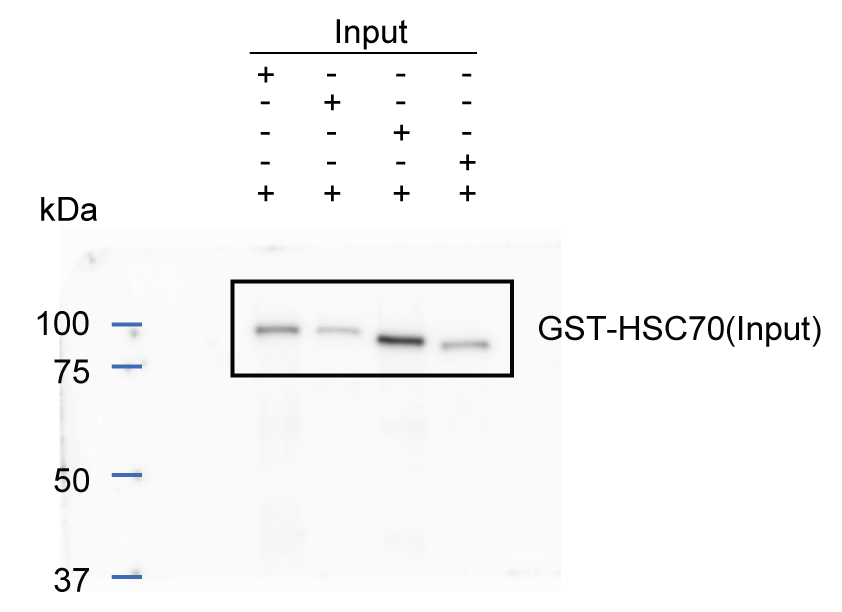

Supplement: Supplementary file 2 — Source data Fig. 1 [file 44319_2025_376_MOESM2_ESM.zip › Fig.1/Fig.1D/GST-HSC70 Input/Fig.1D GST-HSC70 (Input).tif]

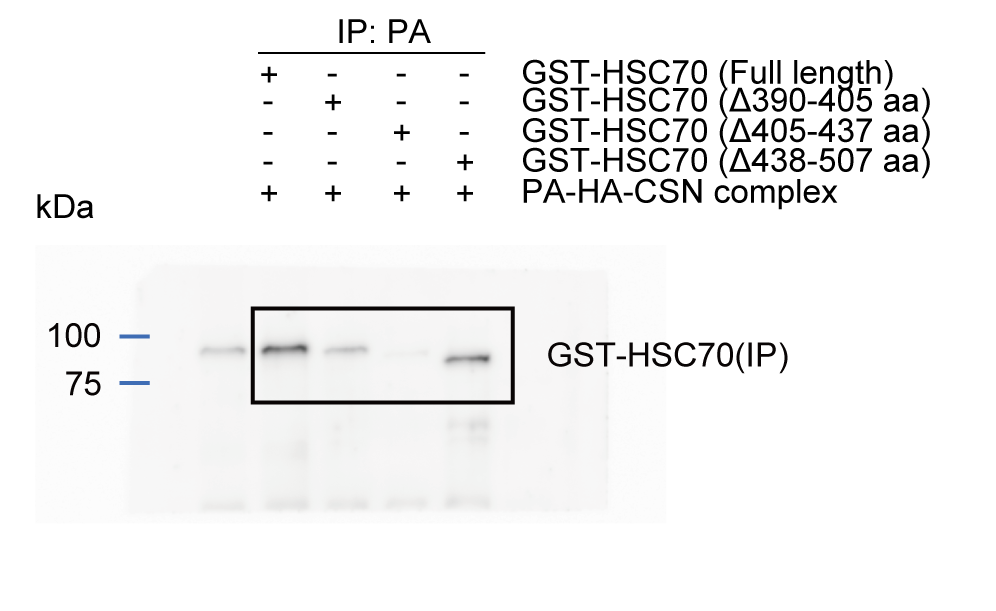

Supplement: Supplementary file 2 — Source data Fig. 1 [file 44319_2025_376_MOESM2_ESM.zip › Fig.1/Fig.1D/GST-HSC70 IP/Fig.1D GST-HSC70 (PA-IP).tif]

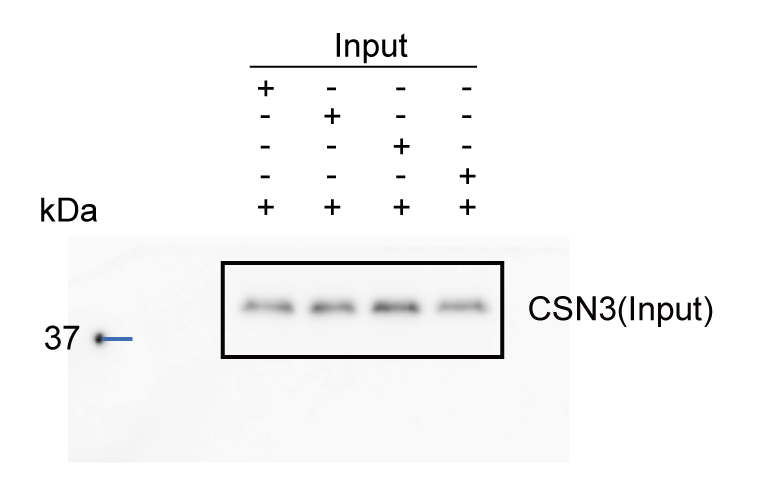

Supplement: Supplementary file 2 — Source data Fig. 1 [file 44319_2025_376_MOESM2_ESM.zip › Fig.1/Fig.1D/PA(CSN3) Input/Fig.1D CSN3 (Input).tif]

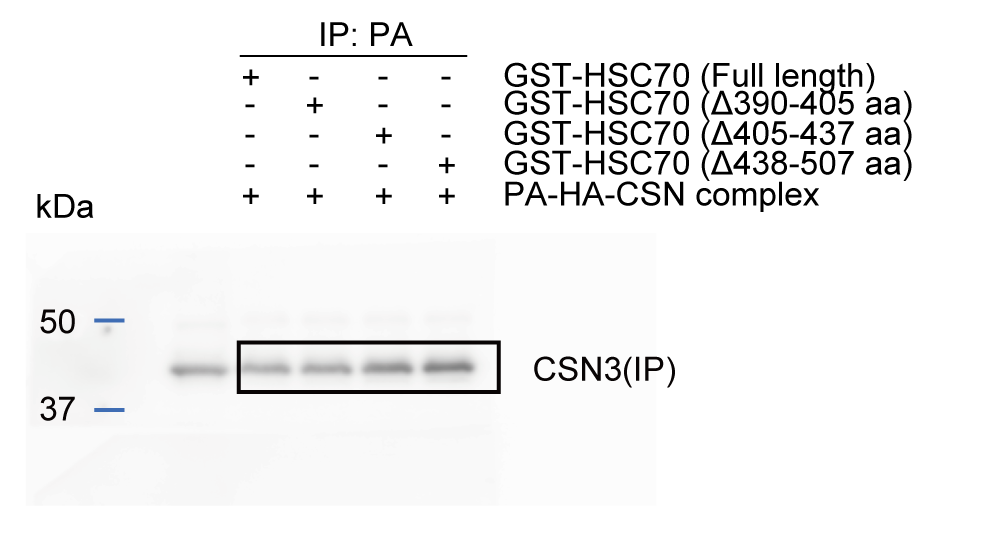

Supplement: Supplementary file 2 — Source data Fig. 1 [file 44319_2025_376_MOESM2_ESM.zip › Fig.1/Fig.1D/PA(CSN3) IP/Fig.1D CSN3 (PA-IP).tif]

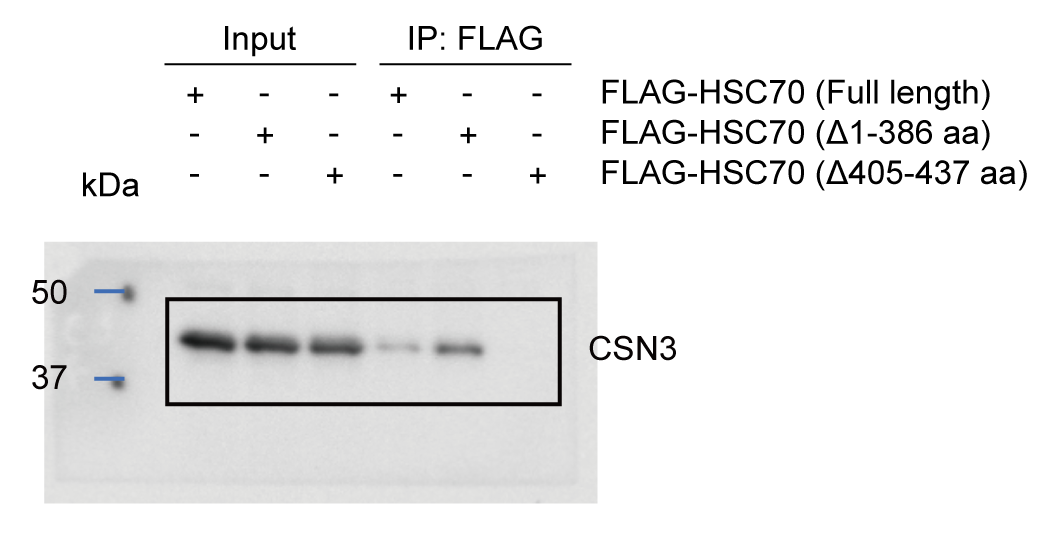

Supplement: Supplementary file 2 — Source data Fig. 1 [file 44319_2025_376_MOESM2_ESM.zip › Fig.1/Fig.1E/CSN3/Fig.1E CSN3.tif]

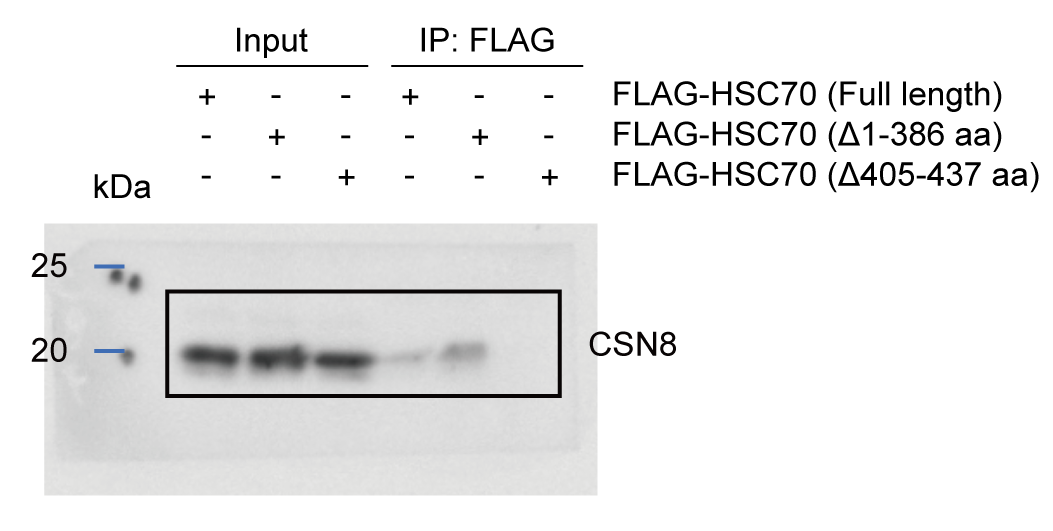

Supplement: Supplementary file 2 — Source data Fig. 1 [file 44319_2025_376_MOESM2_ESM.zip › Fig.1/Fig.1E/CSN8/Fig.1E CSN8.tif]

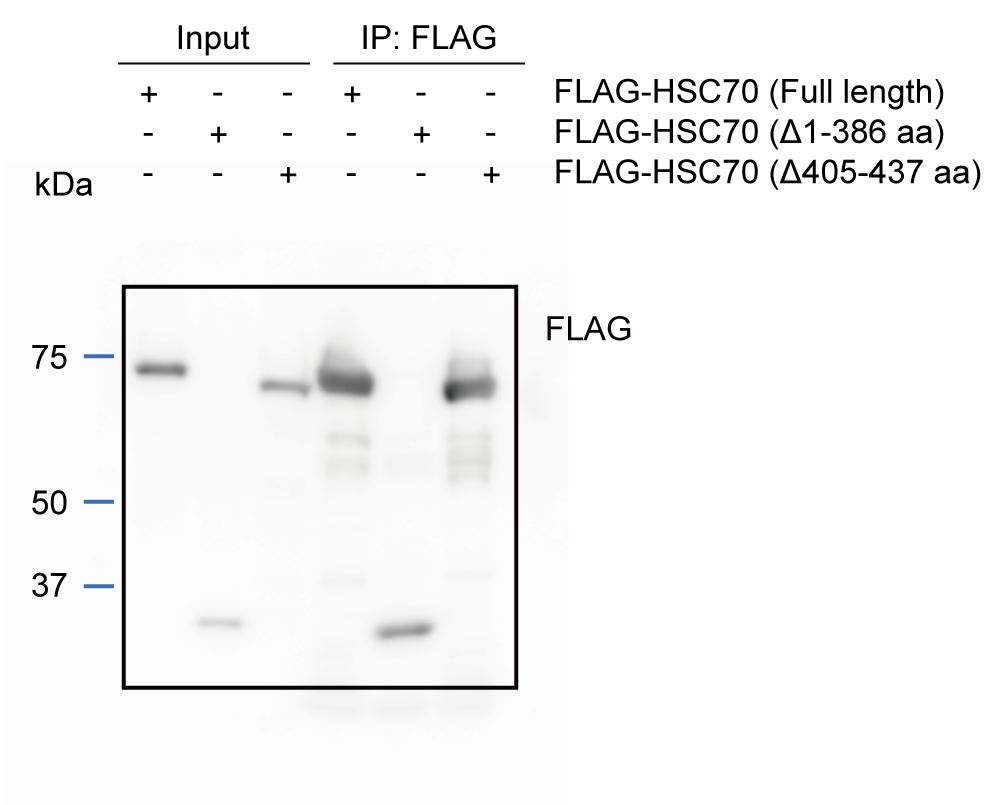

Supplement: Supplementary file 2 — Source data Fig. 1 [file 44319_2025_376_MOESM2_ESM.zip › Fig.1/Fig.1E/FLAG-HSC70/Fig.1E FLAG-HSC70.tif]

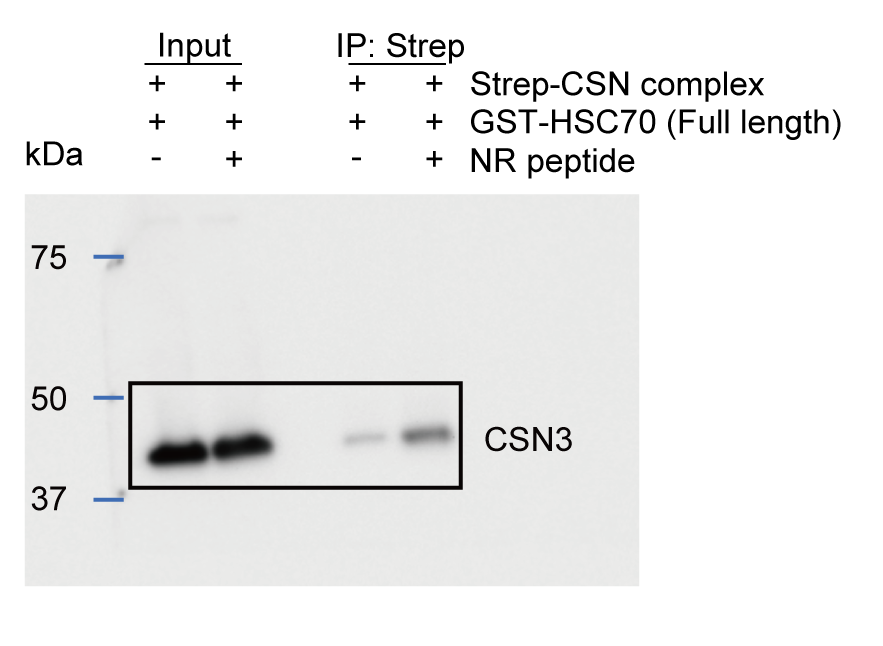

Supplement: Supplementary file 2 — Source data Fig. 1 [file 44319_2025_376_MOESM2_ESM.zip › Fig.1/Fig.1F/CSN3/Fig.1F CSN3.tif]

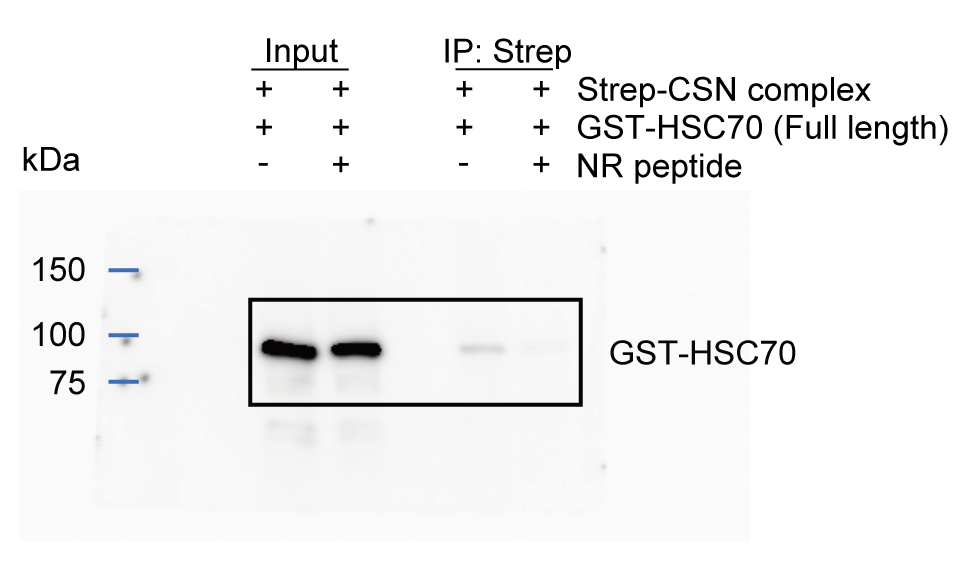

Supplement: Supplementary file 2 — Source data Fig. 1 [file 44319_2025_376_MOESM2_ESM.zip › Fig.1/Fig.1F/GST-HSC70/Fig.1F GST-HSC70.tif]

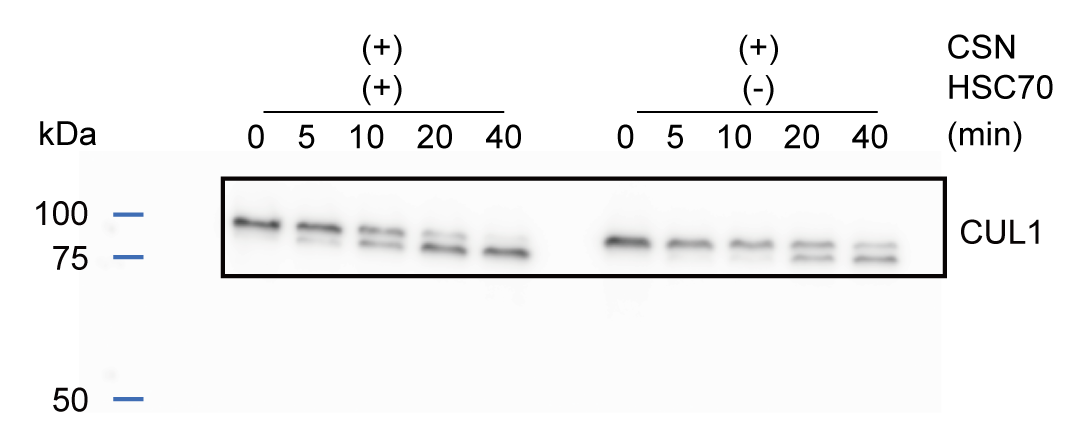

Supplement: Supplementary file 3 — Source data Fig. 2 [file 44319_2025_376_MOESM3_ESM.zip › Fig.2/Fig.2A/CUL1/Fig. 2A CUL1.tif]

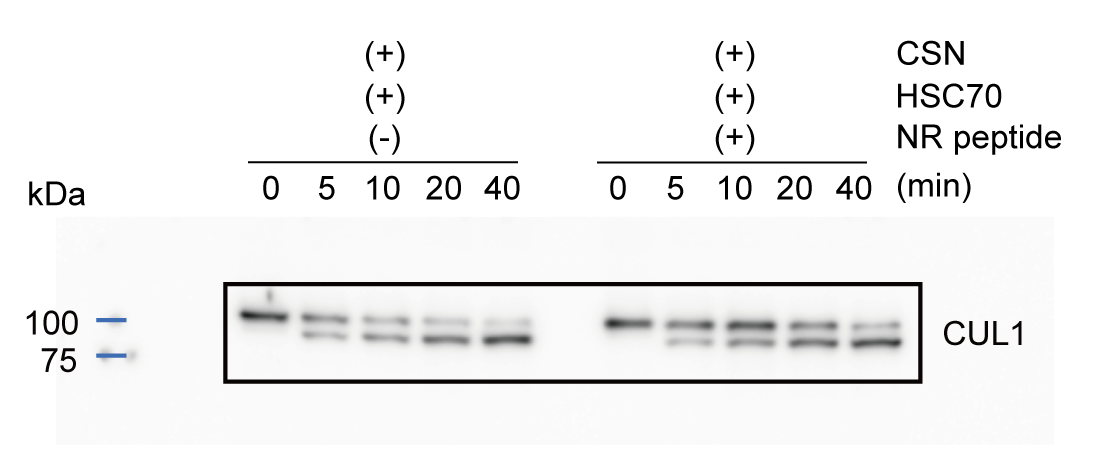

Supplement: Supplementary file 3 — Source data Fig. 2 [file 44319_2025_376_MOESM3_ESM.zip › Fig.2/Fig.2B/CUL1/Fig. 2B CUL1.tif]

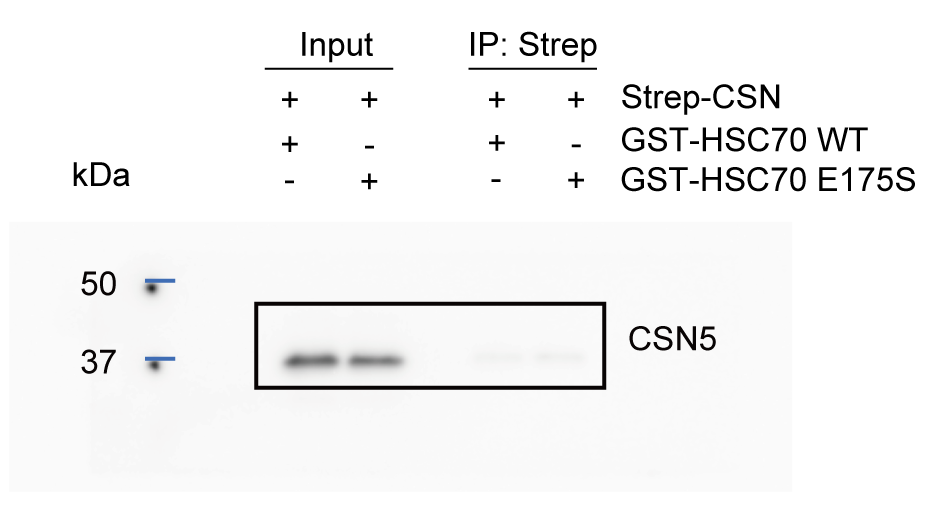

Supplement: Supplementary file 3 — Source data Fig. 2 [file 44319_2025_376_MOESM3_ESM.zip › Fig.2/Fig.2C/CSN5/Fig.2C CSN5.tif]

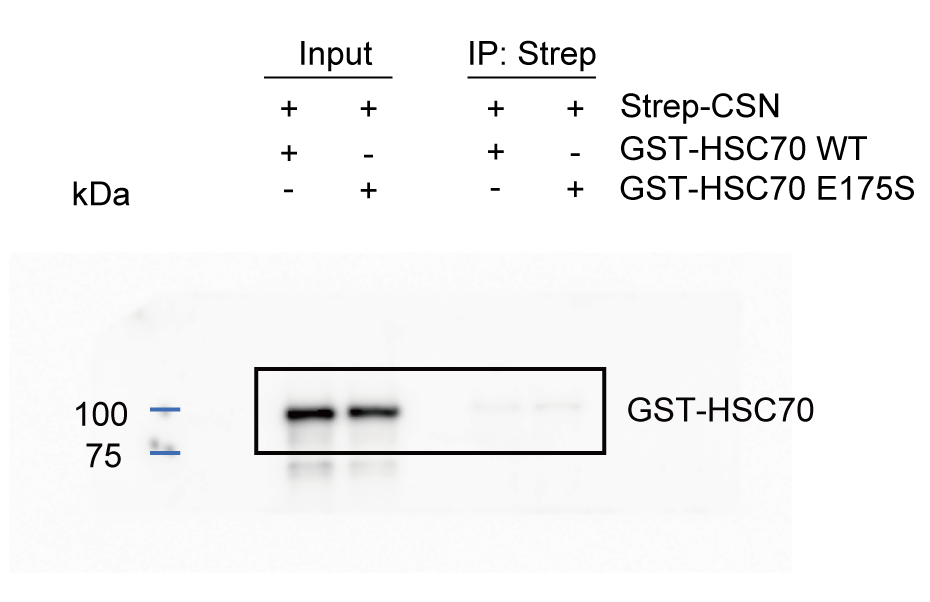

Supplement: Supplementary file 3 — Source data Fig. 2 [file 44319_2025_376_MOESM3_ESM.zip › Fig.2/Fig.2C/GST-HSC70/Fig. 2C GST-HSC70.tif]

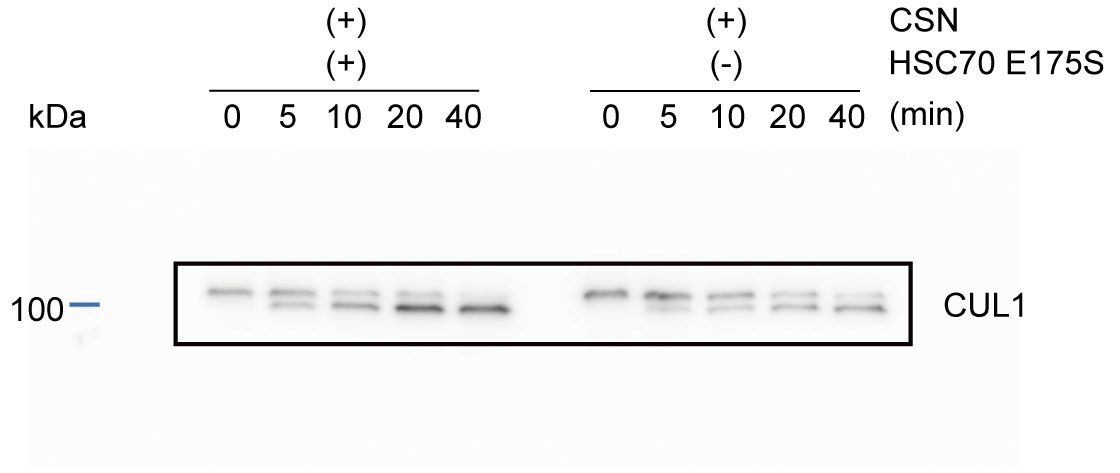

Supplement: Supplementary file 3 — Source data Fig. 2 [file 44319_2025_376_MOESM3_ESM.zip › Fig.2/Fig.2D/CUL1/Fig.2D CUL1.tif]

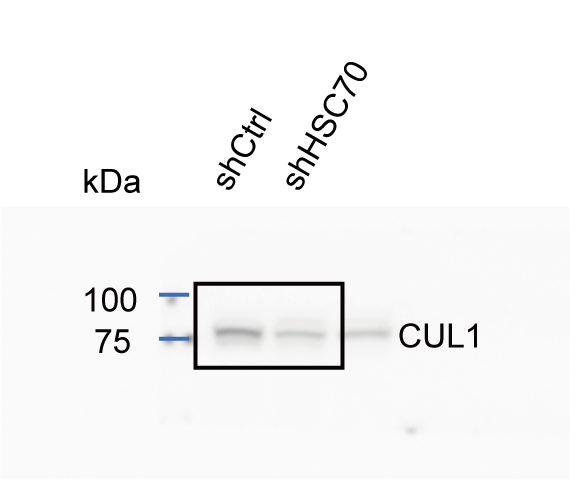

Supplement: Supplementary file 3 — Source data Fig. 2 [file 44319_2025_376_MOESM3_ESM.zip › Fig.2/Fig.2E/CUL1/Fig.2E CUL1.tif]

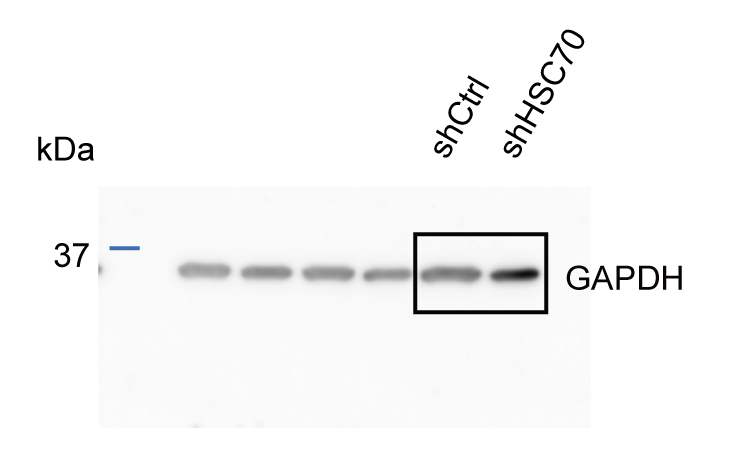

Supplement: Supplementary file 3 — Source data Fig. 2 [file 44319_2025_376_MOESM3_ESM.zip › Fig.2/Fig.2E/GAPDH/Fig.2E GAPDH.tif]

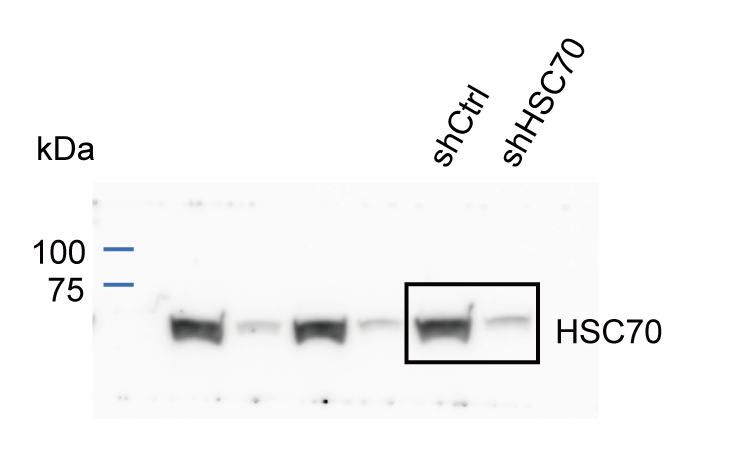

Supplement: Supplementary file 3 — Source data Fig. 2 [file 44319_2025_376_MOESM3_ESM.zip › Fig.2/Fig.2E/HSC70/Fig.2E HSC70.tif]

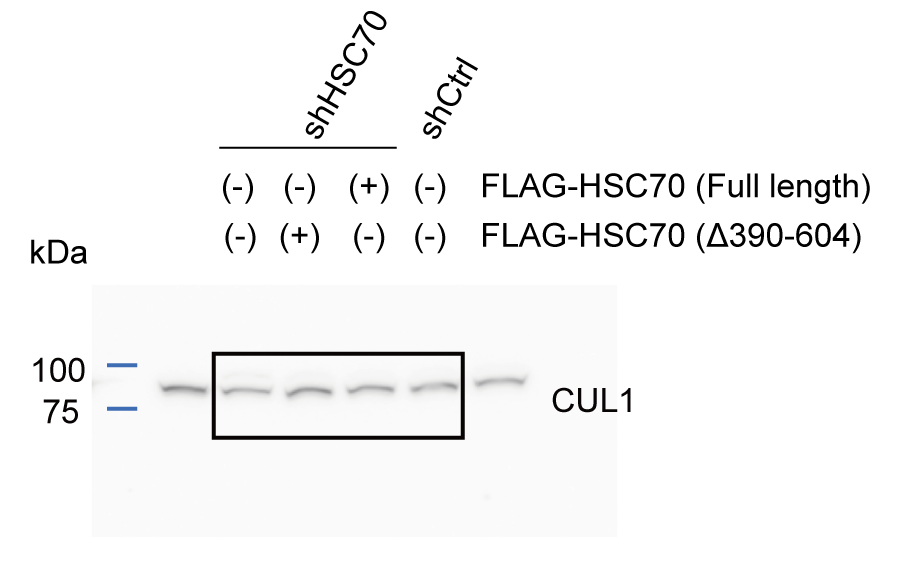

Supplement: Supplementary file 3 — Source data Fig. 2 [file 44319_2025_376_MOESM3_ESM.zip › Fig.2/Fig.2F/CUL1/Fig.2F CUL1.tif]

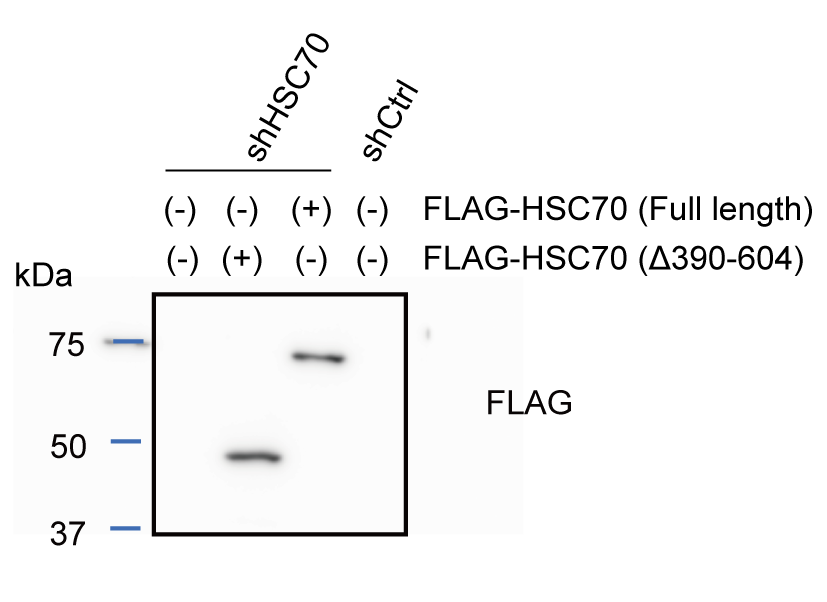

Supplement: Supplementary file 3 — Source data Fig. 2 [file 44319_2025_376_MOESM3_ESM.zip › Fig.2/Fig.2F/FLAG/Fig.2F FLAG.tif]

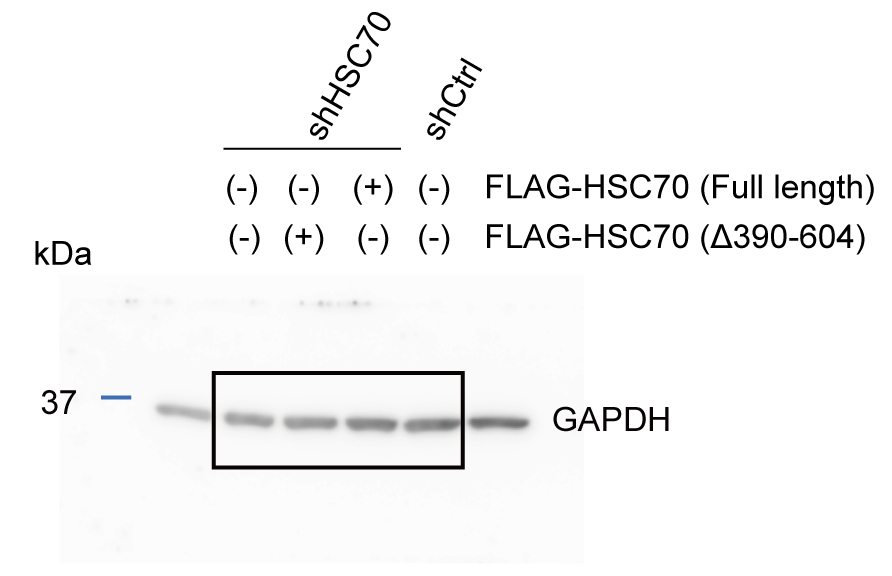

Supplement: Supplementary file 3 — Source data Fig. 2 [file 44319_2025_376_MOESM3_ESM.zip › Fig.2/Fig.2F/GAPDH/Fig.2F GAPDH.tif]

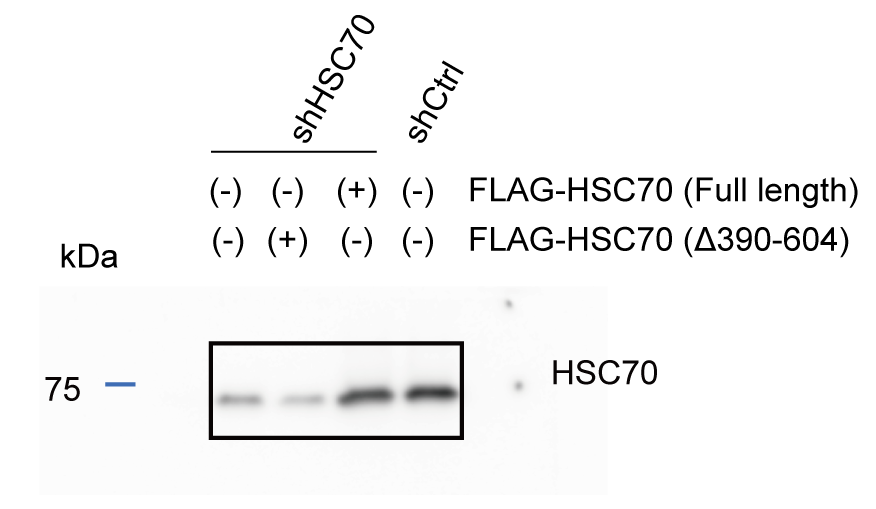

Supplement: Supplementary file 3 — Source data Fig. 2 [file 44319_2025_376_MOESM3_ESM.zip › Fig.2/Fig.2F/HSC70/Fig.2F HSC70.tif]

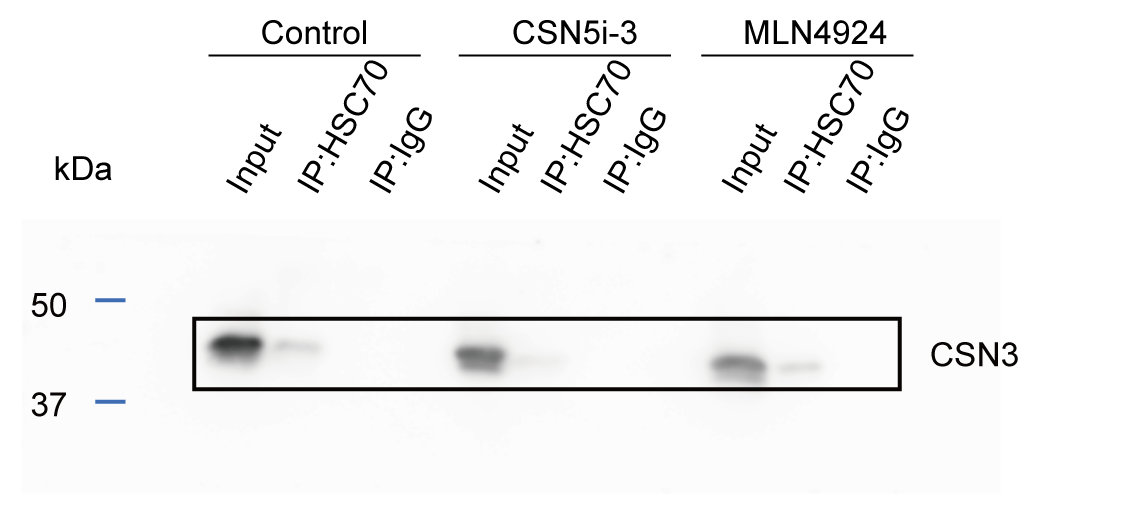

Supplement: Supplementary file 4 — Source data Fig. 3 [file 44319_2025_376_MOESM4_ESM.zip › Fig.3/Fig.3A/CSN3/Fig.3A CSN3.tif]

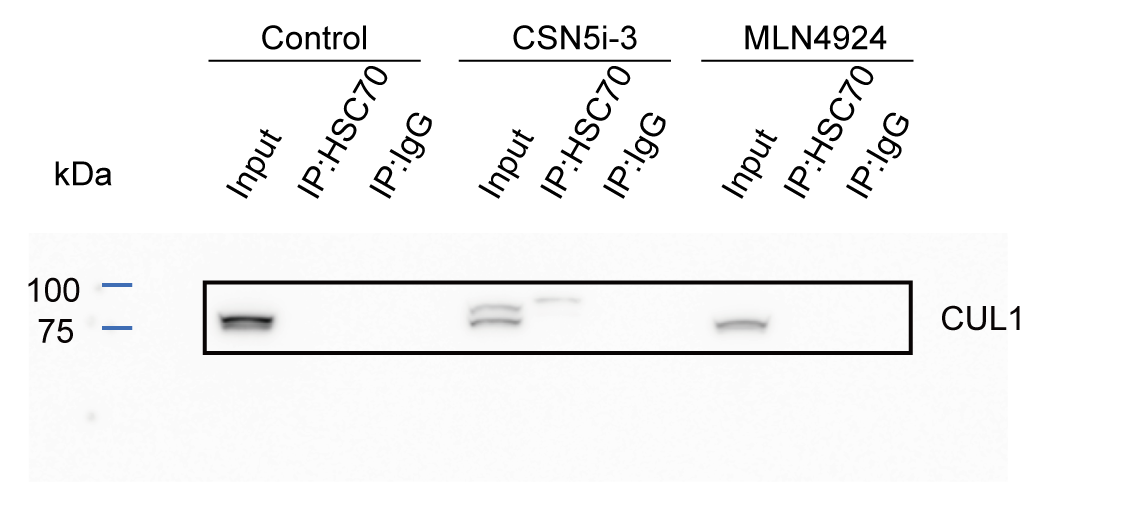

Supplement: Supplementary file 4 — Source data Fig. 3 [file 44319_2025_376_MOESM4_ESM.zip › Fig.3/Fig.3A/CUL1/Fig.3A CUL1.tif]

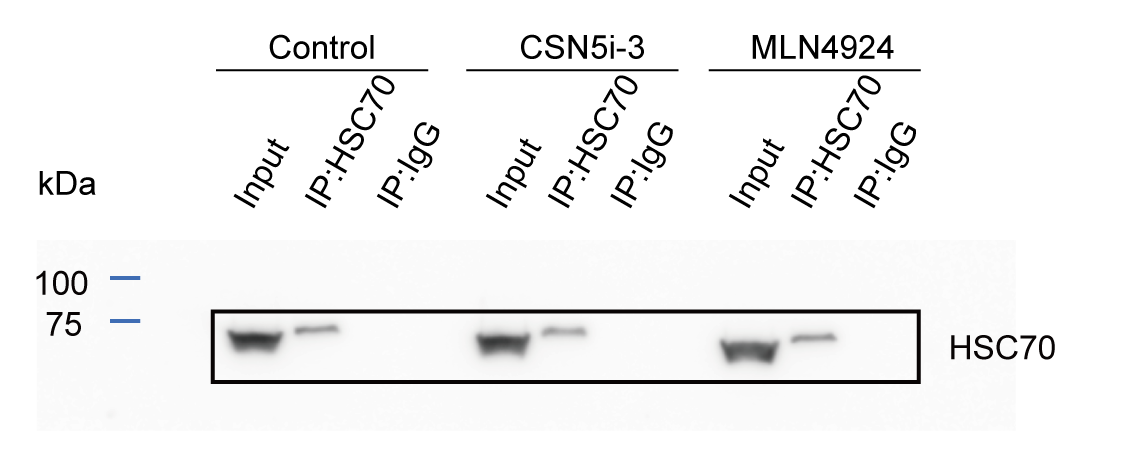

Supplement: Supplementary file 4 — Source data Fig. 3 [file 44319_2025_376_MOESM4_ESM.zip › Fig.3/Fig.3A/HSC70/Fig. 3A HSC70.tif]

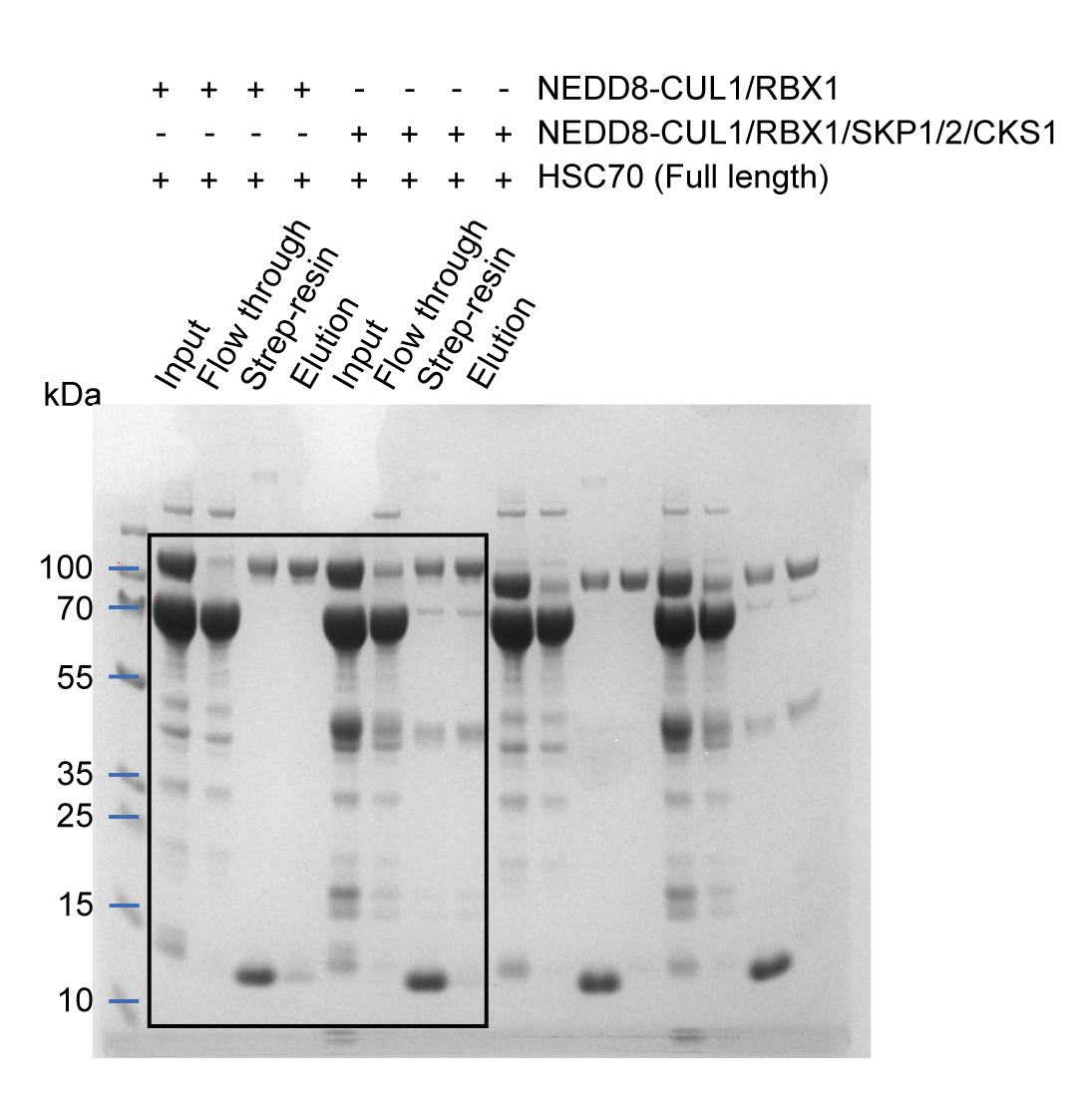

Supplement: Supplementary file 4 — Source data Fig. 3 [file 44319_2025_376_MOESM4_ESM.zip › Fig.3/Fig.3B/Fig. 3B.tif]

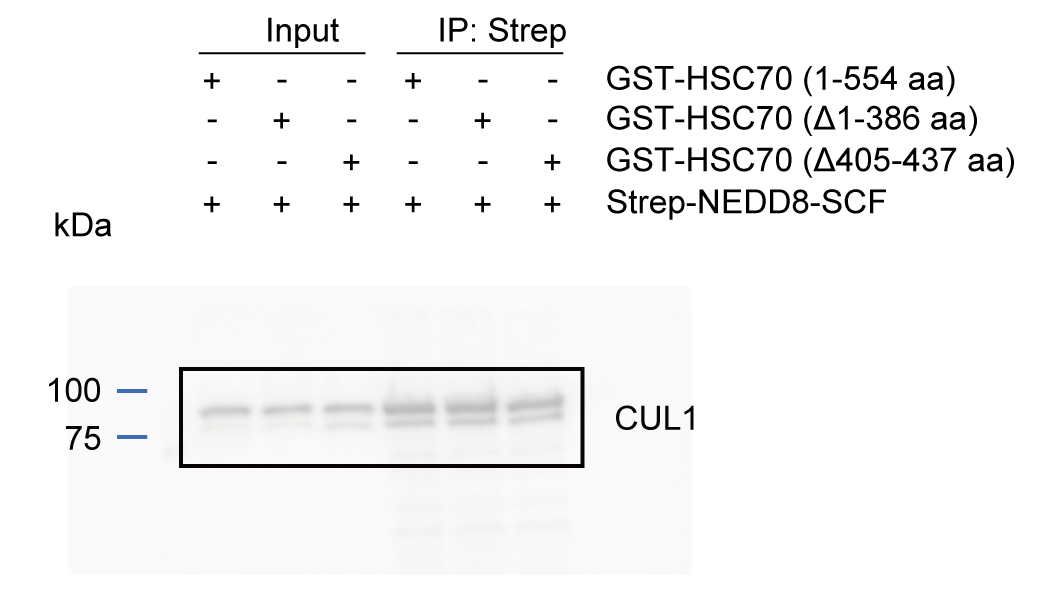

Supplement: Supplementary file 4 — Source data Fig. 3 [file 44319_2025_376_MOESM4_ESM.zip › Fig.3/Fig.3C/CUL1/Fig. 3C CUL1.tif]

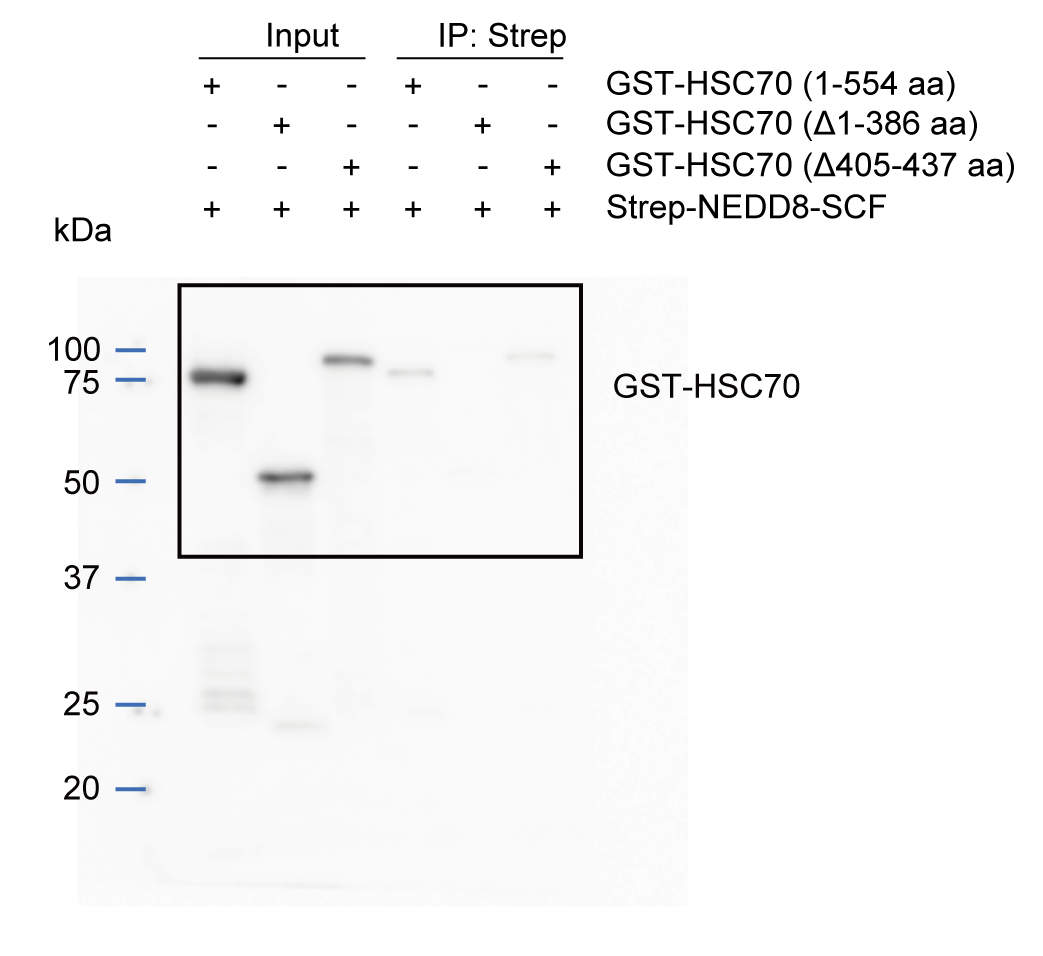

Supplement: Supplementary file 4 — Source data Fig. 3 [file 44319_2025_376_MOESM4_ESM.zip › Fig.3/Fig.3C/GST/Fig.3C GST-HSC70.tif]

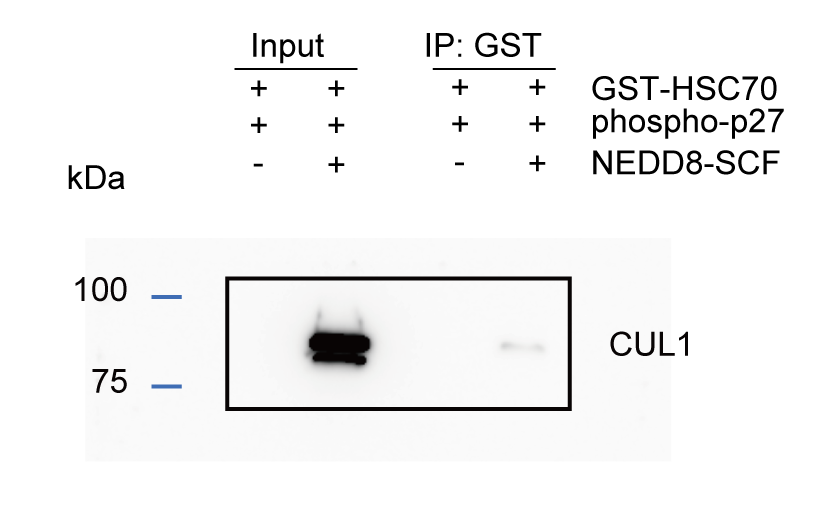

Supplement: Supplementary file 5 — Source data Fig. 4 [file 44319_2025_376_MOESM5_ESM.zip › Fig.4/Fig.4A/CUL1/Fig.4A CUL1.tif]

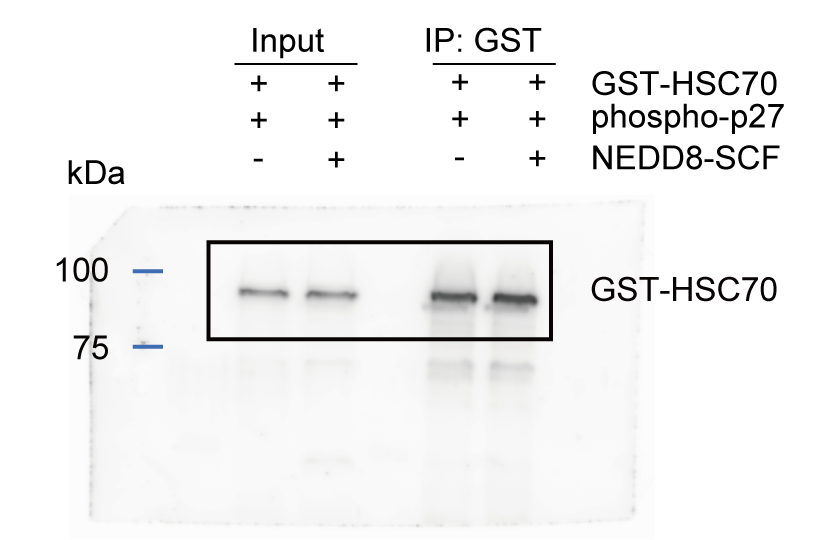

Supplement: Supplementary file 5 — Source data Fig. 4 [file 44319_2025_376_MOESM5_ESM.zip › Fig.4/Fig.4A/GST-HSC70/Fig.4A GST-HSC70.tif]

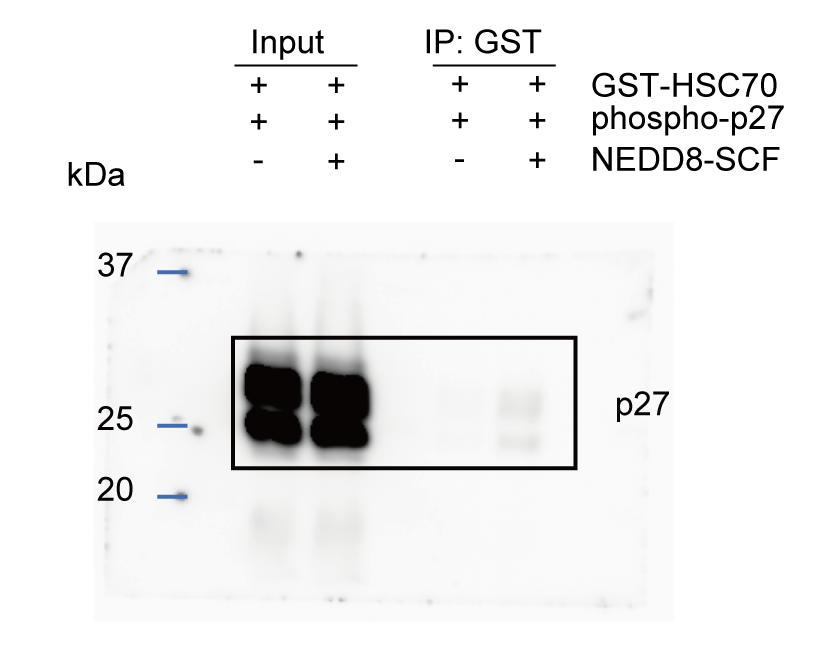

Supplement: Supplementary file 5 — Source data Fig. 4 [file 44319_2025_376_MOESM5_ESM.zip › Fig.4/Fig.4A/p27/Fig.4A p27.tif]

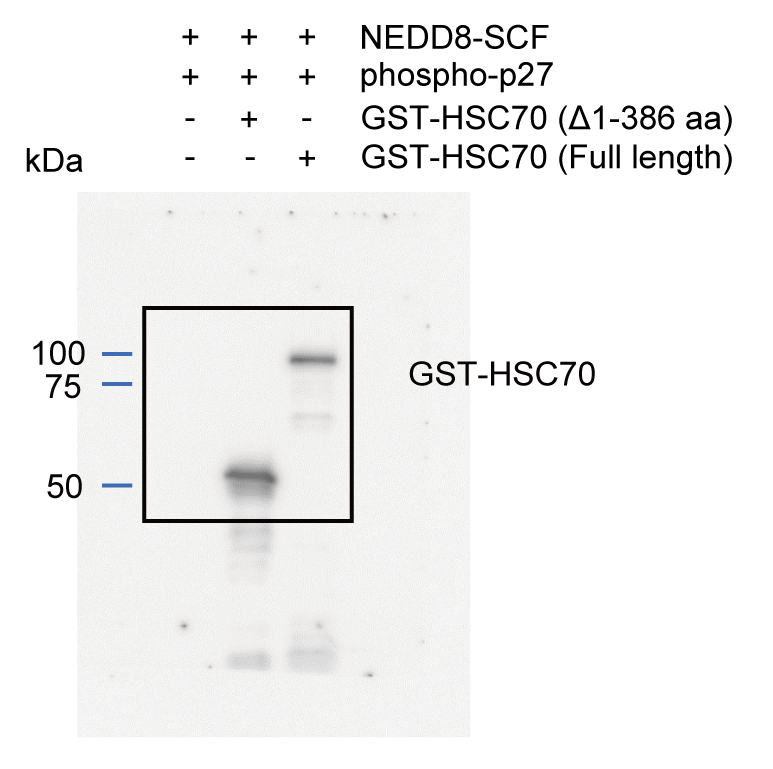

Supplement: Supplementary file 5 — Source data Fig. 4 [file 44319_2025_376_MOESM5_ESM.zip › Fig.4/Fig.4B/GST-HSC70/Fig.4B GST-HSC70.tif]

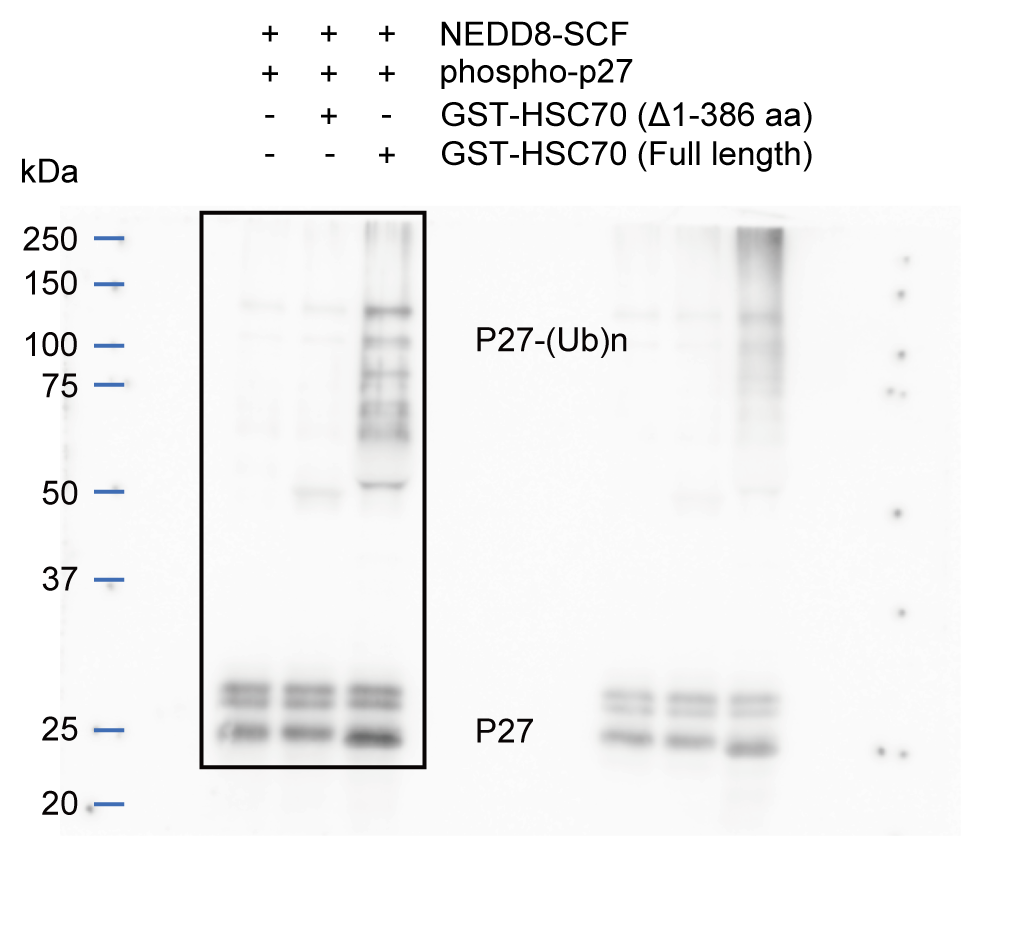

Supplement: Supplementary file 5 — Source data Fig. 4 [file 44319_2025_376_MOESM5_ESM.zip › Fig.4/Fig.4B/p27/Fig.4B p27.tif]

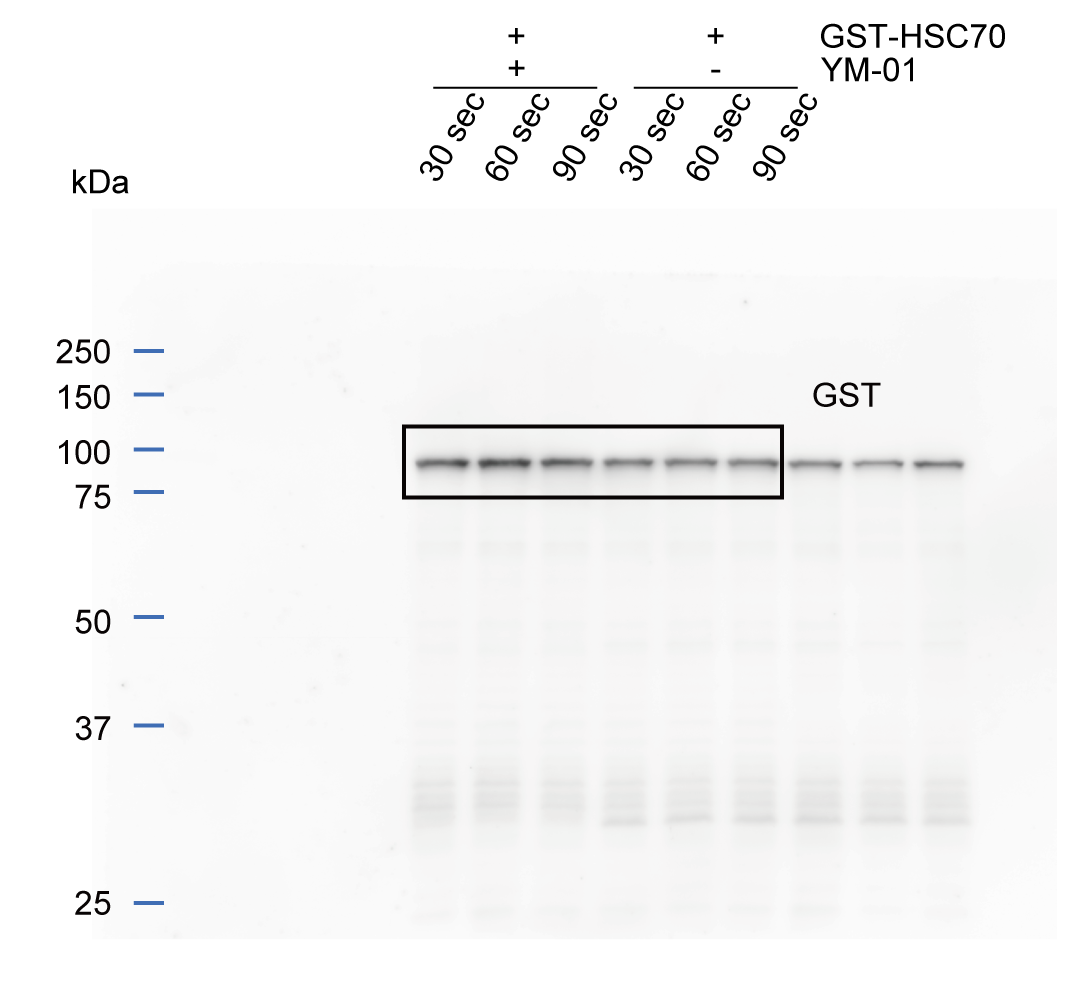

Supplement: Supplementary file 5 — Source data Fig. 4 [file 44319_2025_376_MOESM5_ESM.zip › Fig.4/Fig.4C/GST-HSC70/Fig.4C GST-HSC70.tif]

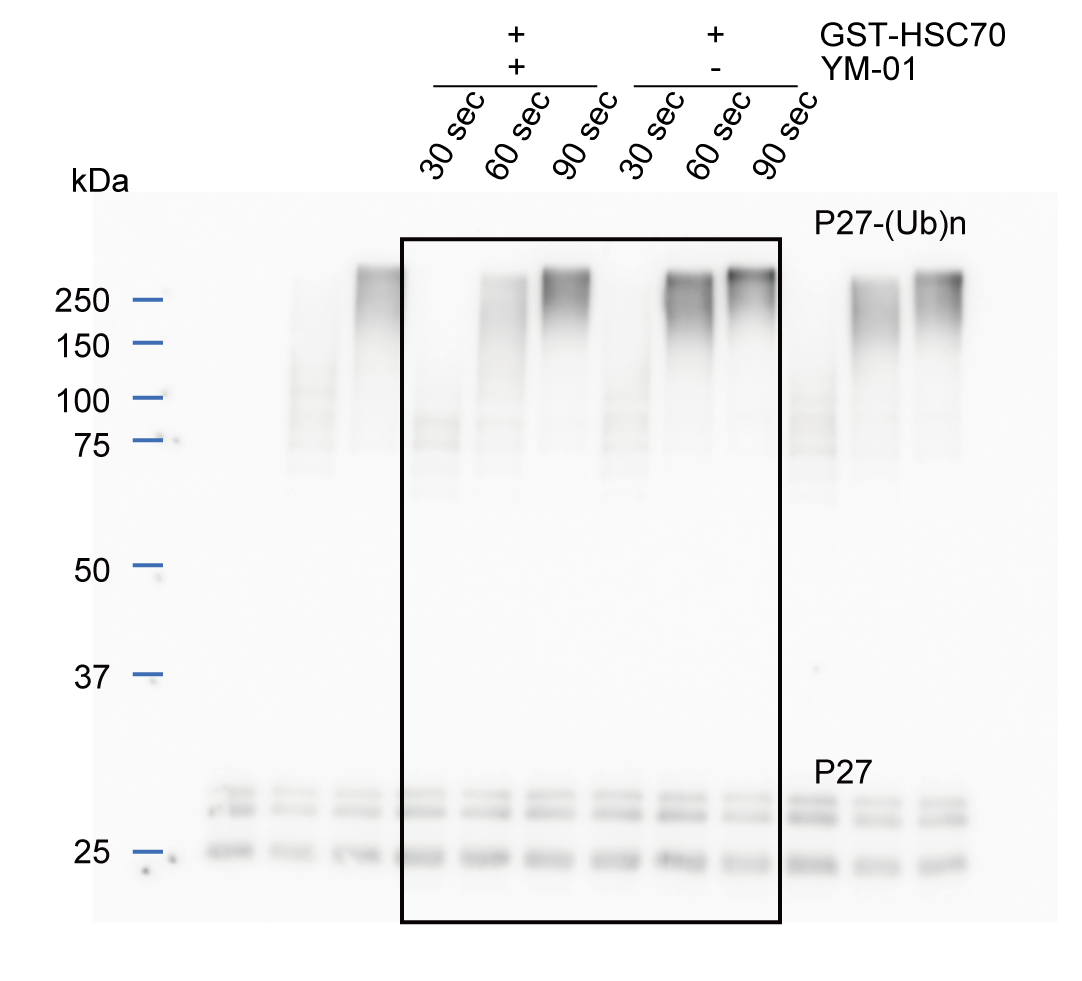

Supplement: Supplementary file 5 — Source data Fig. 4 [file 44319_2025_376_MOESM5_ESM.zip › Fig.4/Fig.4C/p27/Fig.4C p27.tif]

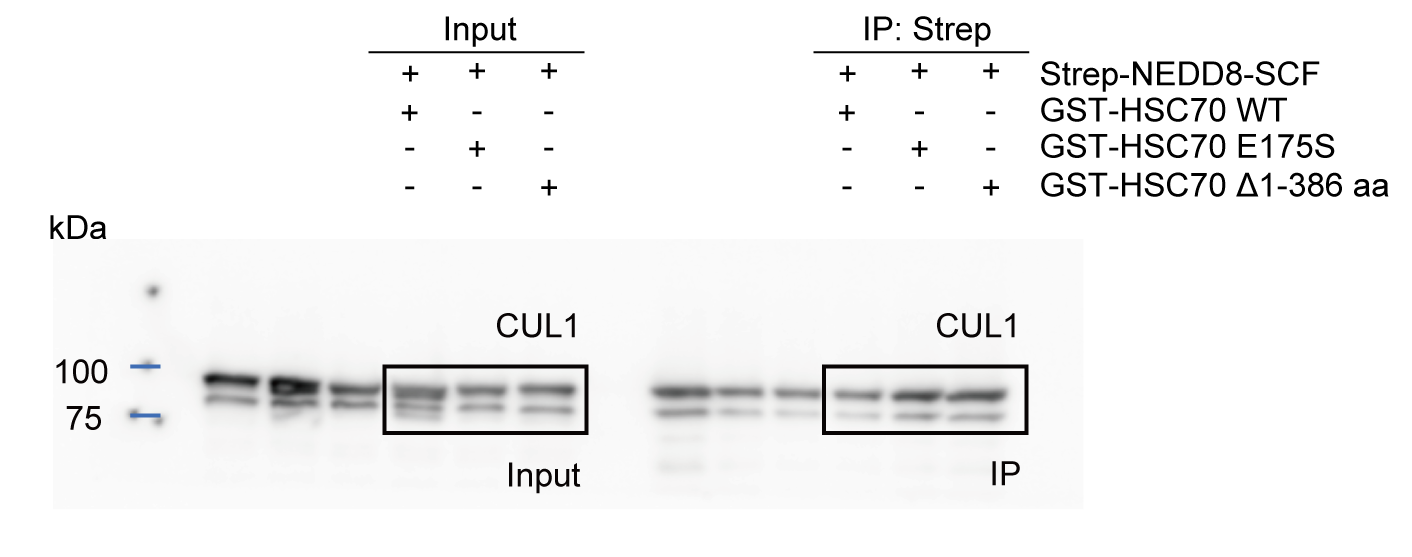

Supplement: Supplementary file 5 — Source data Fig. 4 [file 44319_2025_376_MOESM5_ESM.zip › Fig.4/Fig.4D/CUL1/Fig.4D CUL1.tif]

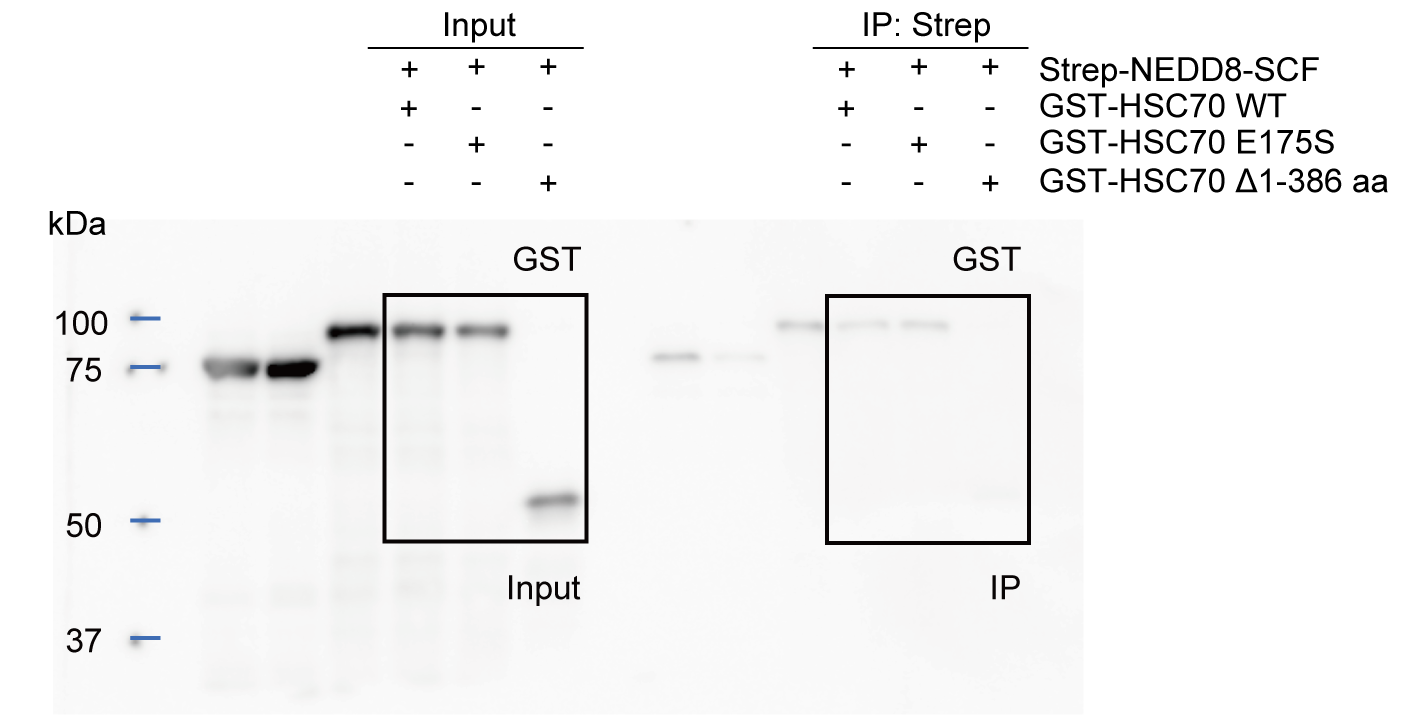

Supplement: Supplementary file 5 — Source data Fig. 4 [file 44319_2025_376_MOESM5_ESM.zip › Fig.4/Fig.4D/GST-HSC70/Fig.4D GST-HSC70.tif]

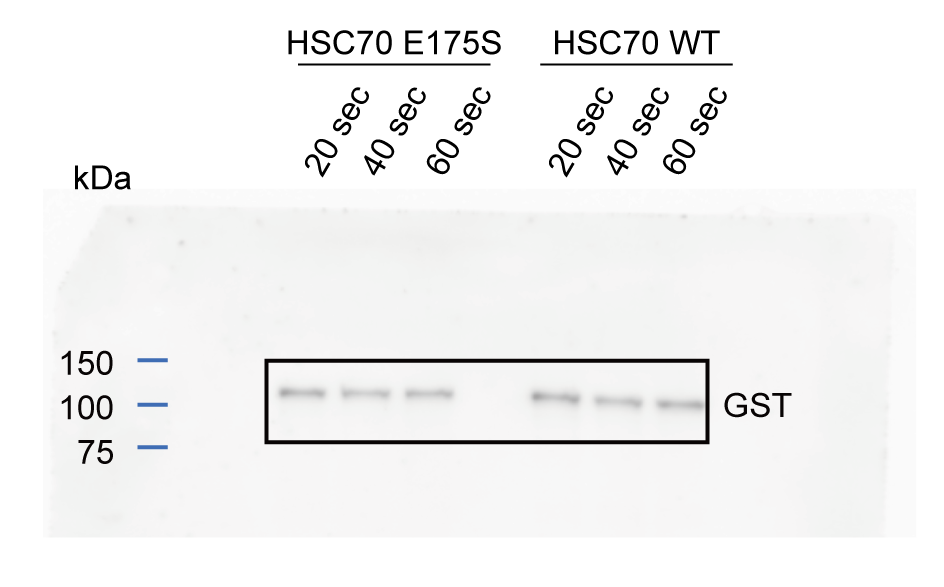

Supplement: Supplementary file 5 — Source data Fig. 4 [file 44319_2025_376_MOESM5_ESM.zip › Fig.4/Fig.4E/GST/Fig.4E GST.tif]

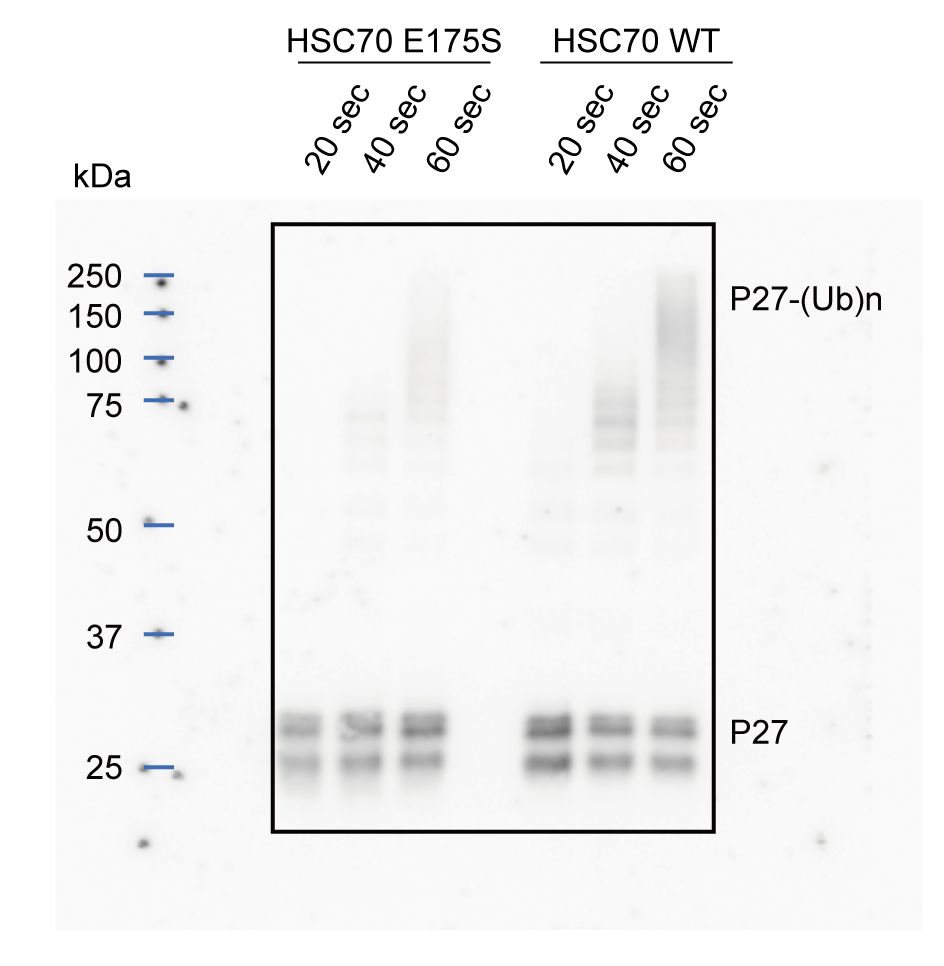

Supplement: Supplementary file 5 — Source data Fig. 4 [file 44319_2025_376_MOESM5_ESM.zip › Fig.4/Fig.4E/p27/Fig.4E p27.tif]

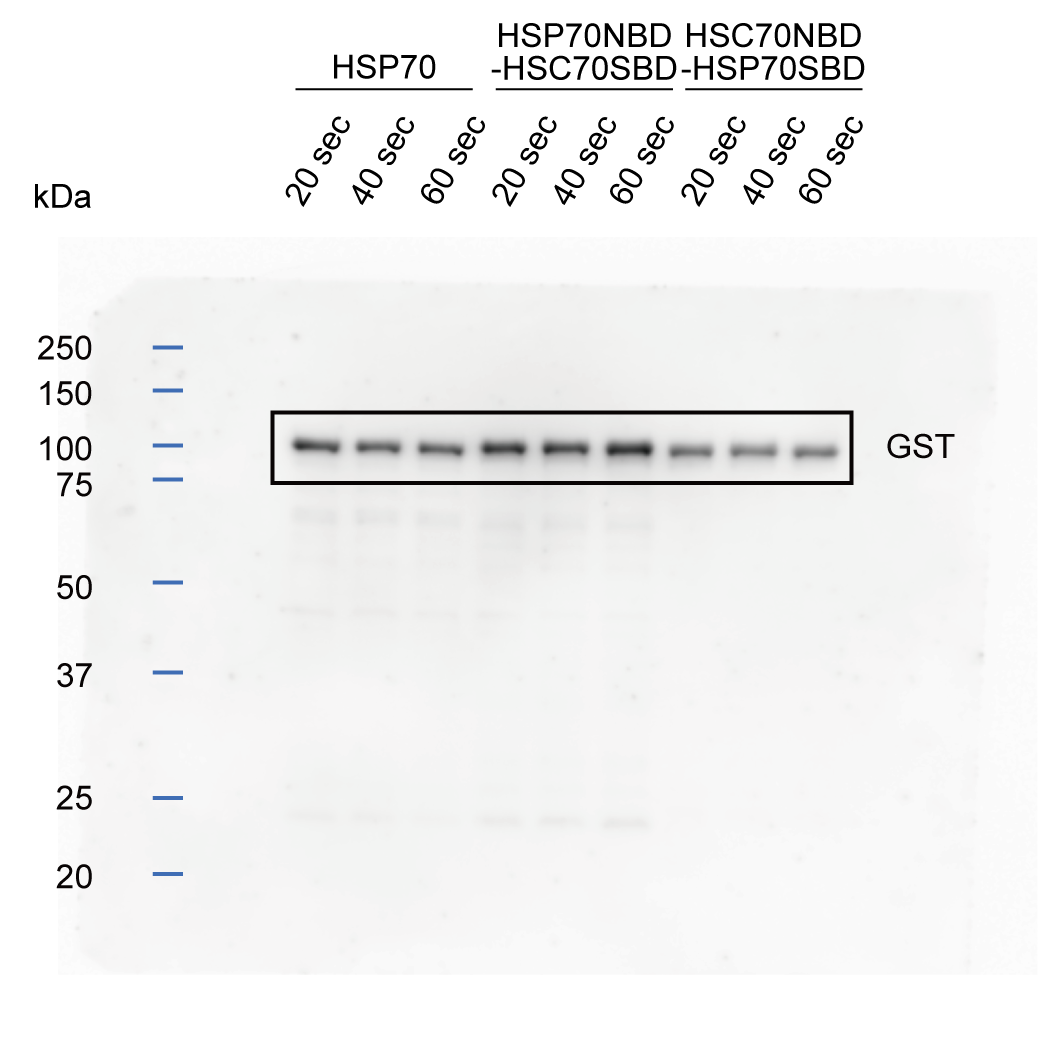

Supplement: Supplementary file 5 — Source data Fig. 4 [file 44319_2025_376_MOESM5_ESM.zip › Fig.4/Fig.4G/GST/Fig.4G GST.tif]

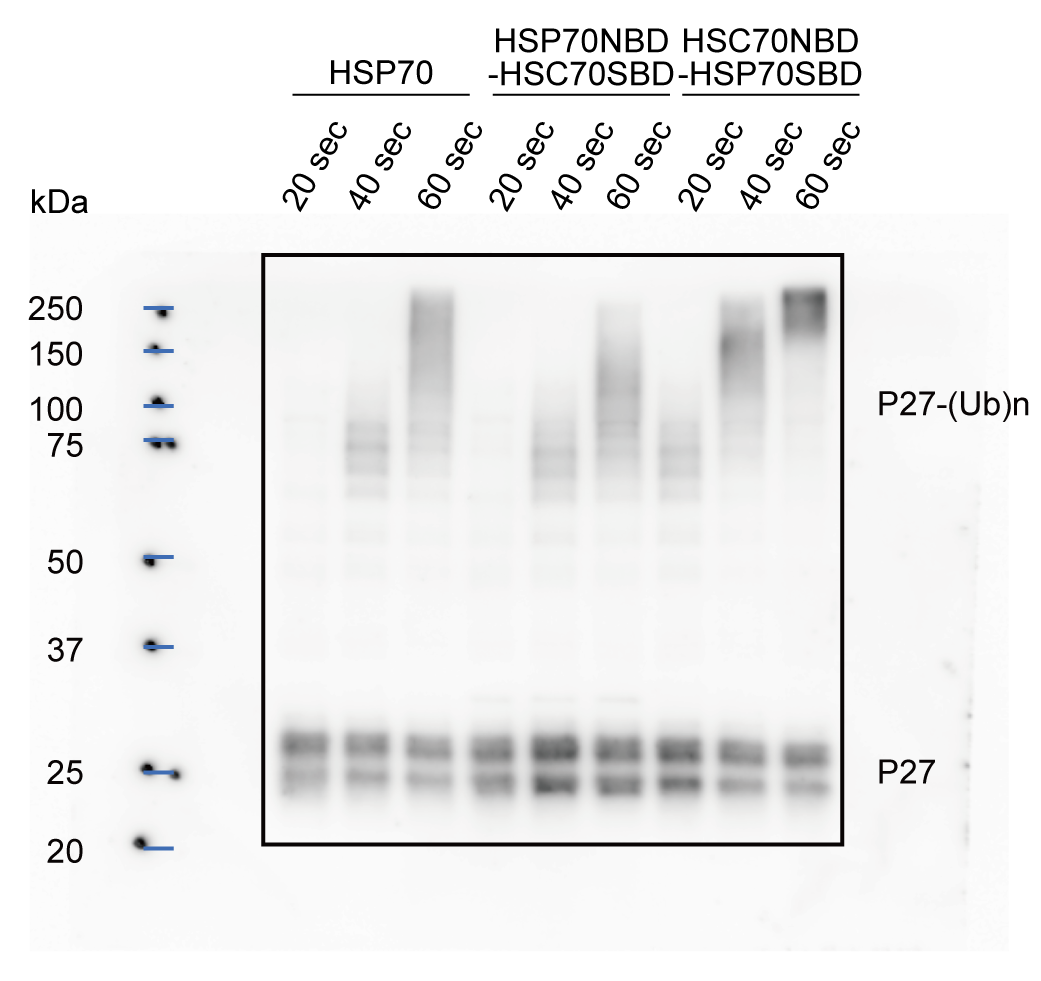

Supplement: Supplementary file 5 — Source data Fig. 4 [file 44319_2025_376_MOESM5_ESM.zip › Fig.4/Fig.4G/p27/Fig.4G p27.tif]

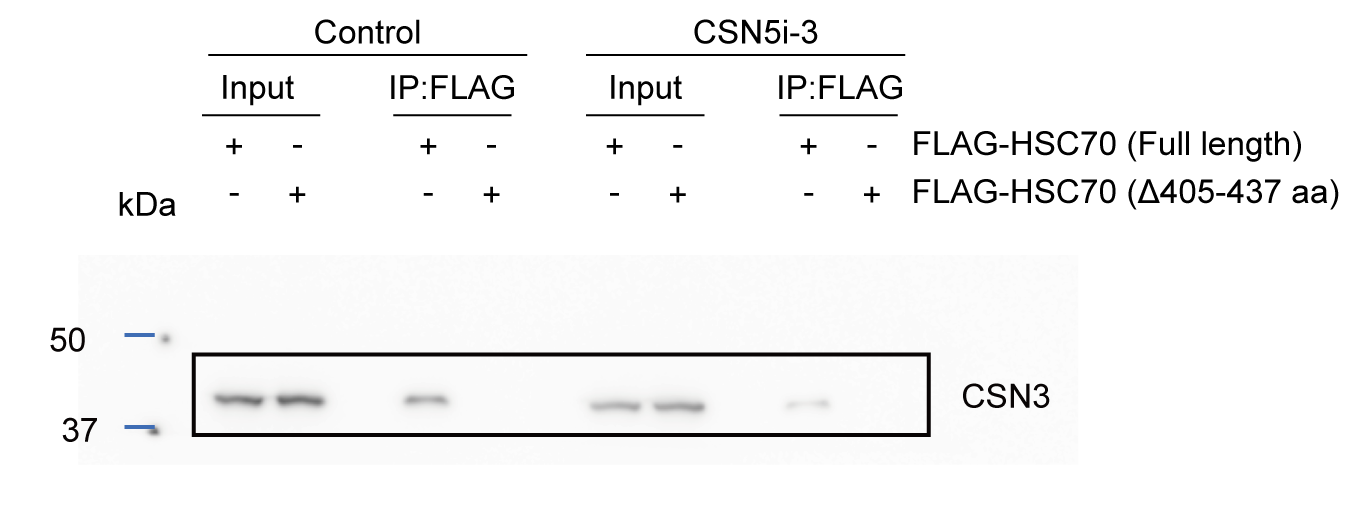

Supplement: Supplementary file 6 — Source data Fig. 5 [file 44319_2025_376_MOESM6_ESM.zip › Fig.5/Fig.5A/CSN3/Fig.5A CSN3.tif]

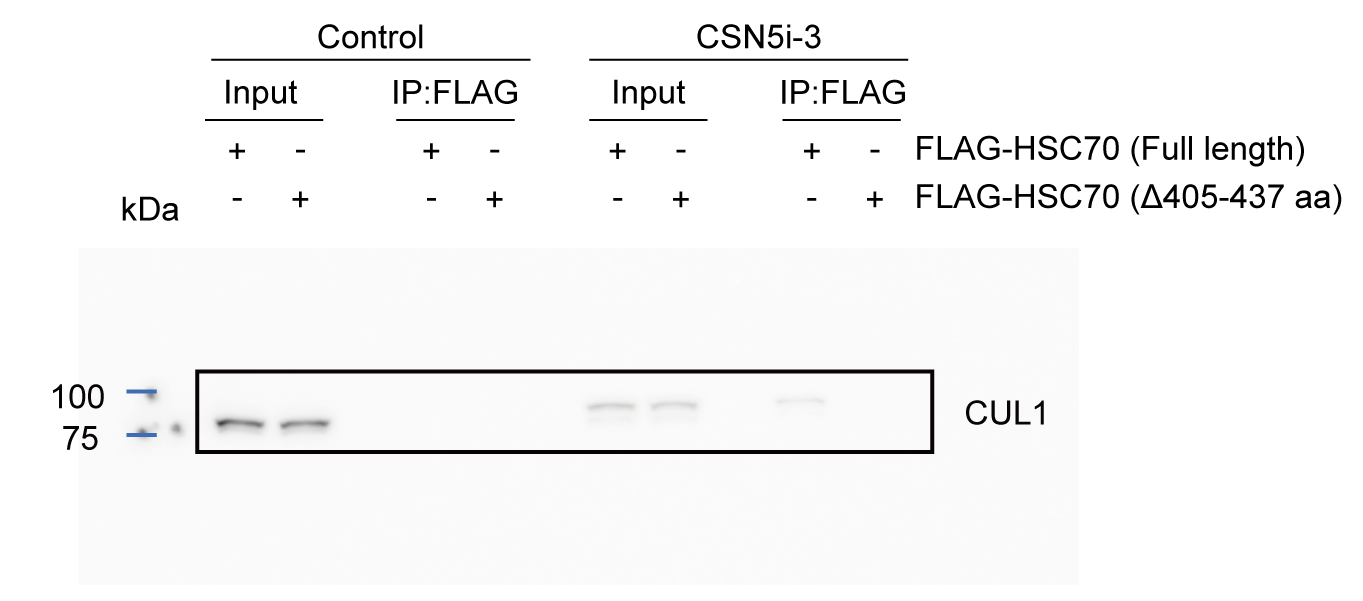

Supplement: Supplementary file 6 — Source data Fig. 5 [file 44319_2025_376_MOESM6_ESM.zip › Fig.5/Fig.5A/CUL1/Fig.5A CUL1.tif]

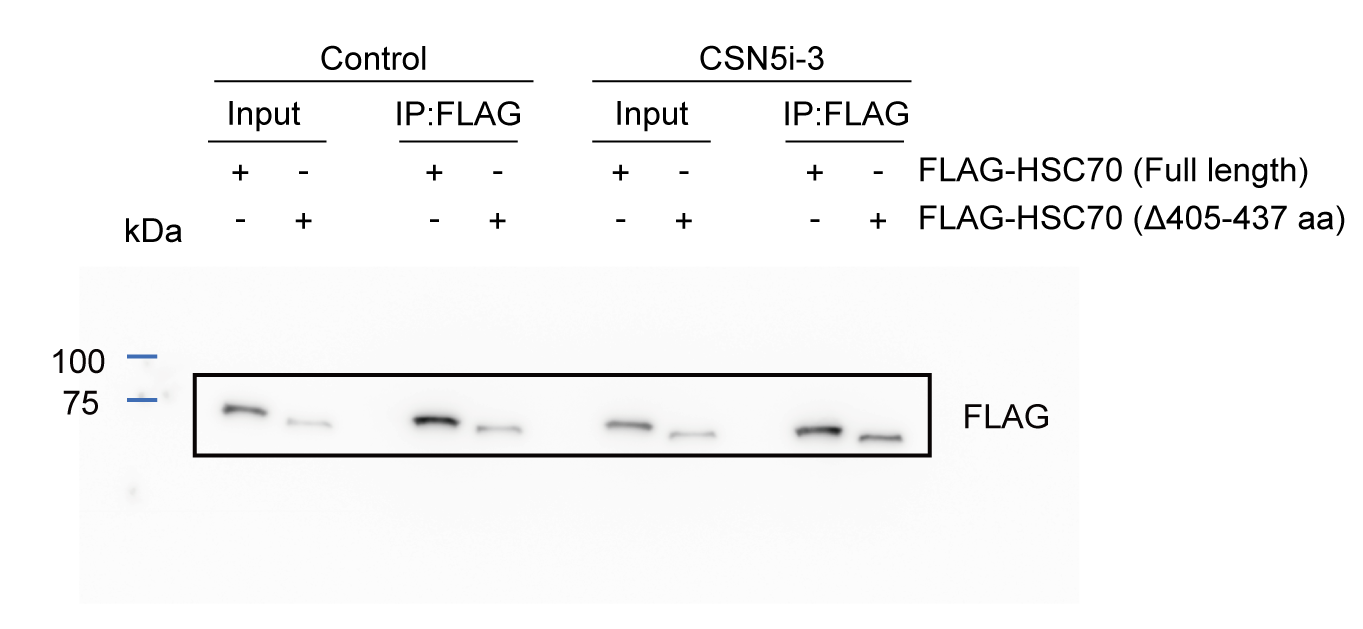

Supplement: Supplementary file 6 — Source data Fig. 5 [file 44319_2025_376_MOESM6_ESM.zip › Fig.5/Fig.5A/FLAG/Fig.5A FLAG.tif]

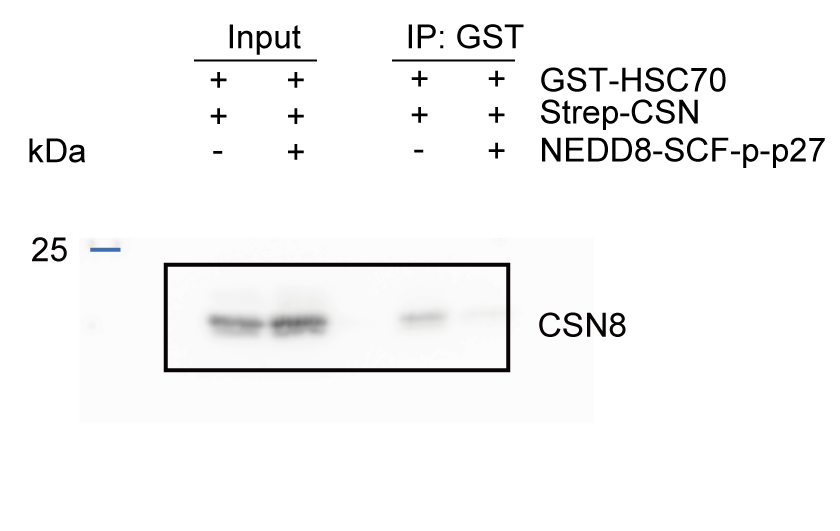

Supplement: Supplementary file 6 — Source data Fig. 5 [file 44319_2025_376_MOESM6_ESM.zip › Fig.5/Fig.5B/CSN8/Fig.5B CSN8.tif]

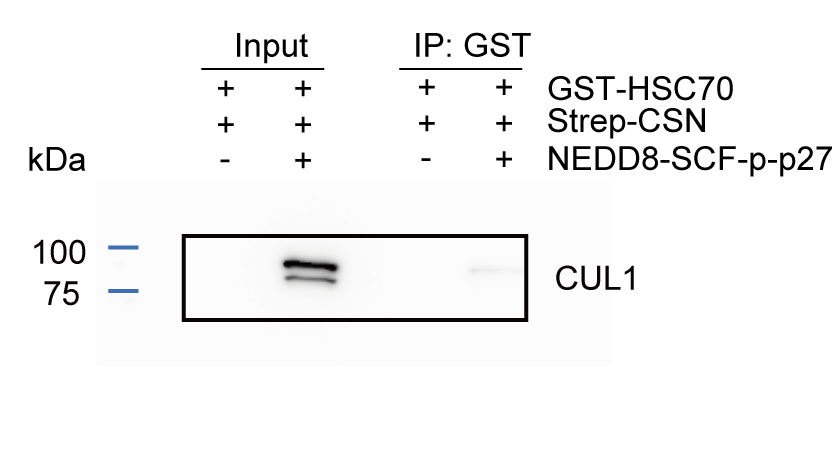

Supplement: Supplementary file 6 — Source data Fig. 5 [file 44319_2025_376_MOESM6_ESM.zip › Fig.5/Fig.5B/CUL1/Fig.5B CUL1.tif]

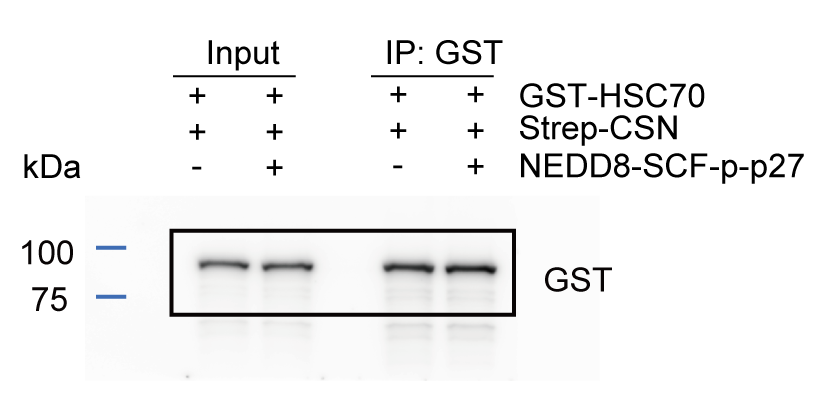

Supplement: Supplementary file 6 — Source data Fig. 5 [file 44319_2025_376_MOESM6_ESM.zip › Fig.5/Fig.5B/GST-HSC70/Fig.5B GST-HSC70.tif]

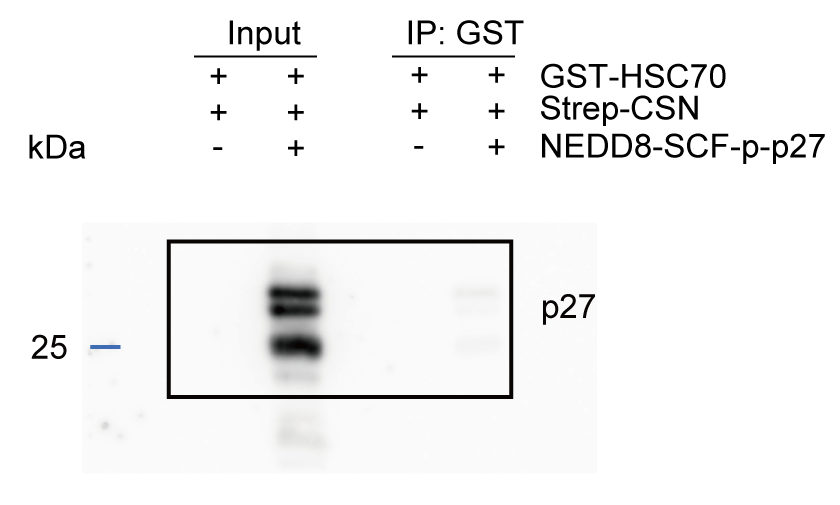

Supplement: Supplementary file 6 — Source data Fig. 5 [file 44319_2025_376_MOESM6_ESM.zip › Fig.5/Fig.5B/p27/Fig.5B p27.tif]

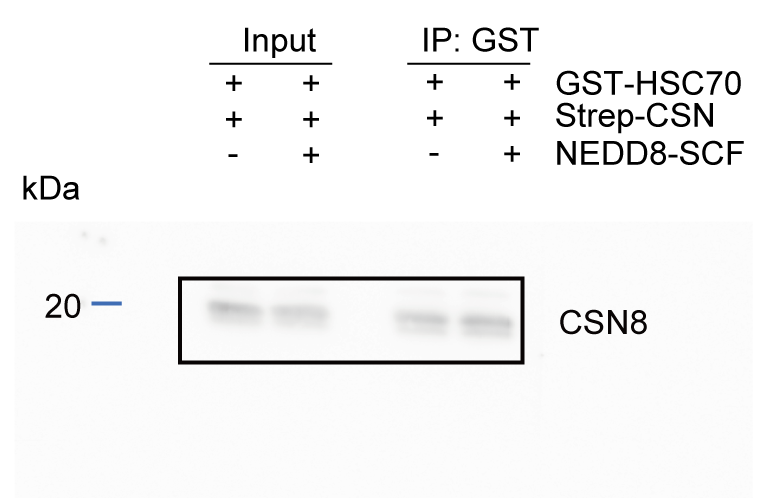

Supplement: Supplementary file 6 — Source data Fig. 5 [file 44319_2025_376_MOESM6_ESM.zip › Fig.5/Fig.5C/CSN8/Fig.5C CSN8.tif]

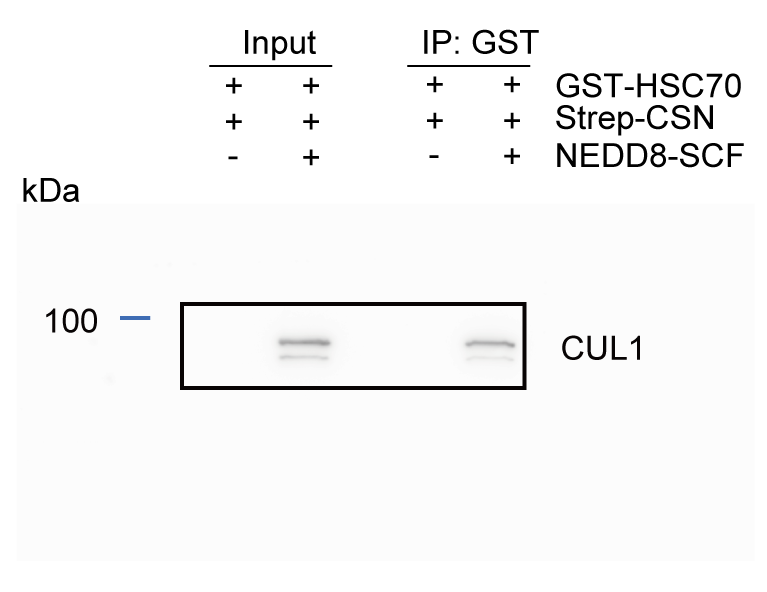

Supplement: Supplementary file 6 — Source data Fig. 5 [file 44319_2025_376_MOESM6_ESM.zip › Fig.5/Fig.5C/CUL1/Fig.5C CUL1.tif]

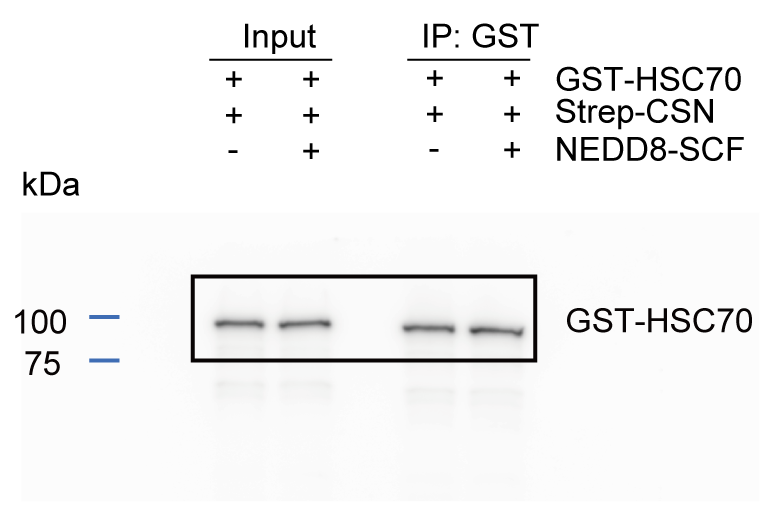

Supplement: Supplementary file 6 — Source data Fig. 5 [file 44319_2025_376_MOESM6_ESM.zip › Fig.5/Fig.5C/GST-HSC70/Fig.5C GST-HSC70.tif]

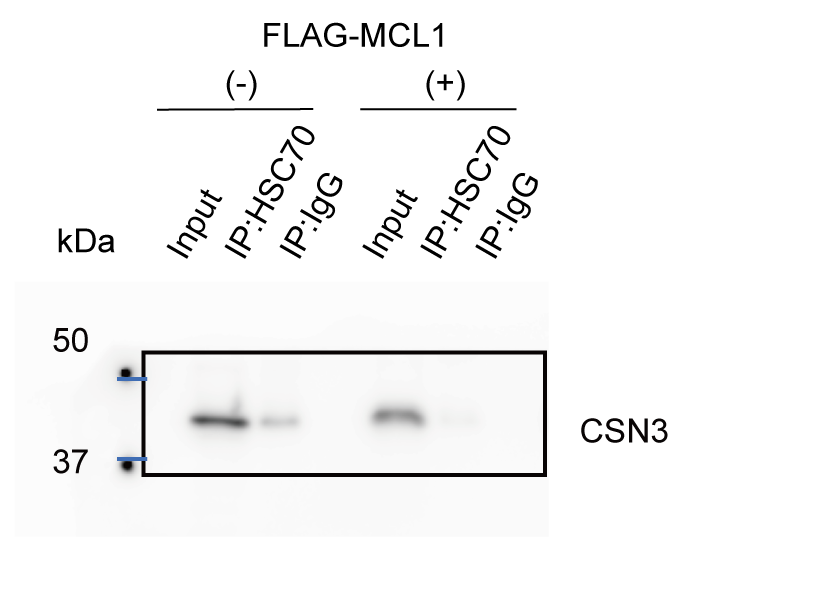

Supplement: Supplementary file 6 — Source data Fig. 5 [file 44319_2025_376_MOESM6_ESM.zip › Fig.5/Fig.5D/CSN3/Fig.5D CSN3.tif]

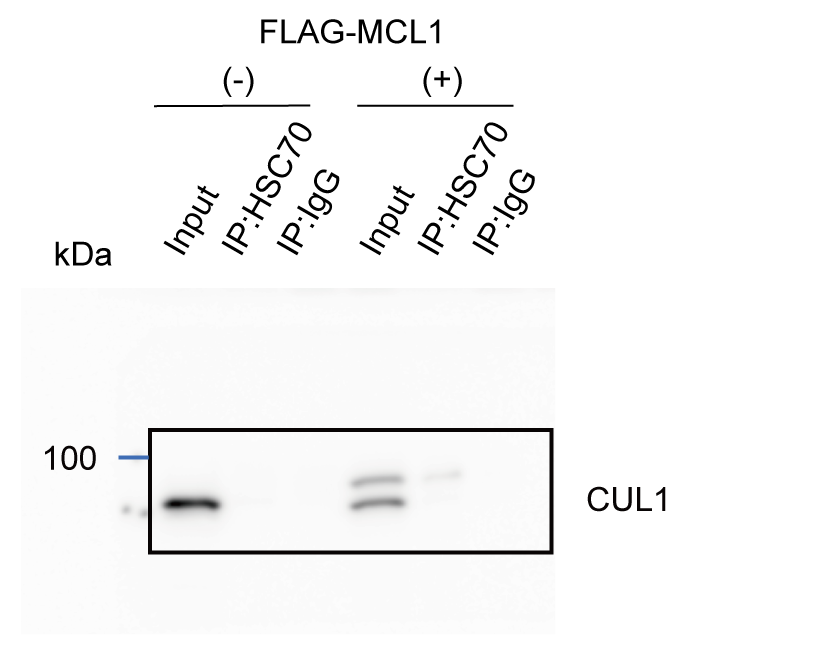

Supplement: Supplementary file 6 — Source data Fig. 5 [file 44319_2025_376_MOESM6_ESM.zip › Fig.5/Fig.5D/CUL1/Fig.5D CUL1.tif]

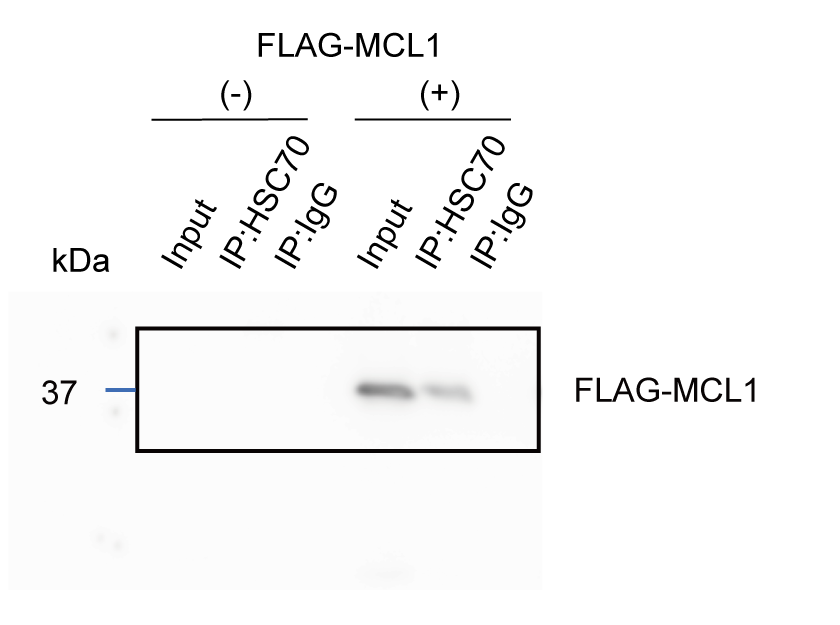

Supplement: Supplementary file 6 — Source data Fig. 5 [file 44319_2025_376_MOESM6_ESM.zip › Fig.5/Fig.5D/FLAG-MCL1/Fig.5D FLAG-MCL1.tif]

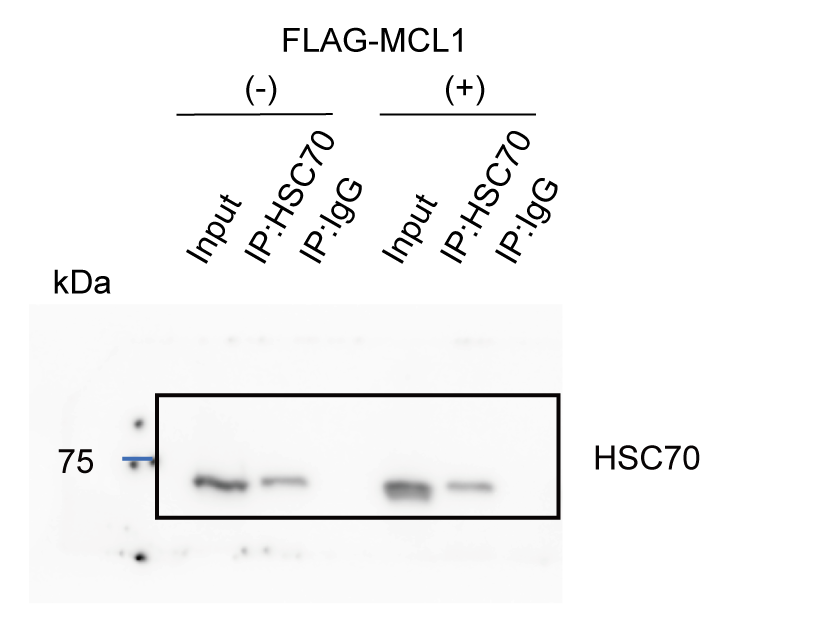

Supplement: Supplementary file 6 — Source data Fig. 5 [file 44319_2025_376_MOESM6_ESM.zip › Fig.5/Fig.5D/HSC70/Fig.5D HSC70.tif]

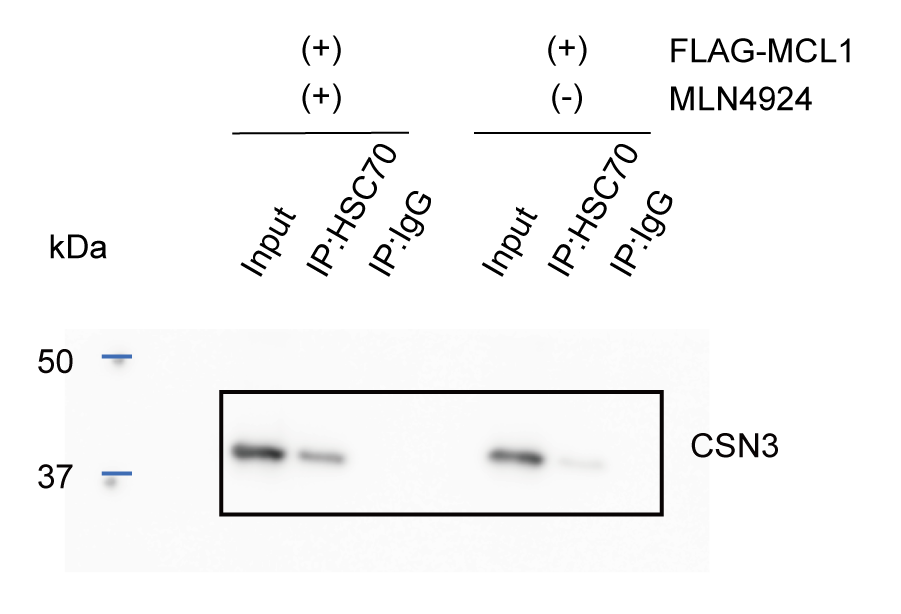

Supplement: Supplementary file 6 — Source data Fig. 5 [file 44319_2025_376_MOESM6_ESM.zip › Fig.5/Fig.5E/CSN3/Fig.5E CSN3.tif]

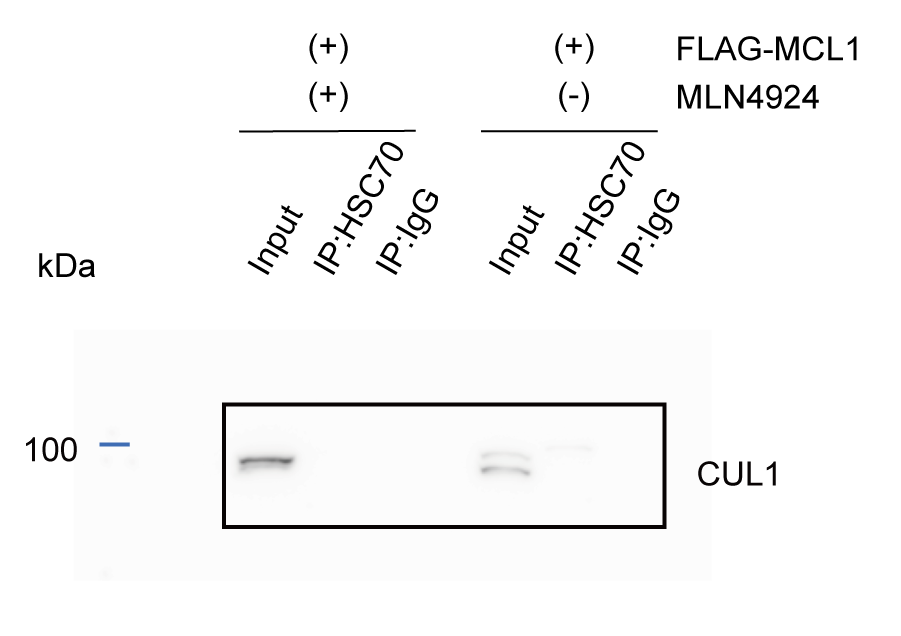

Supplement: Supplementary file 6 — Source data Fig. 5 [file 44319_2025_376_MOESM6_ESM.zip › Fig.5/Fig.5E/CUL1/Fig.5E CUL1.tif]

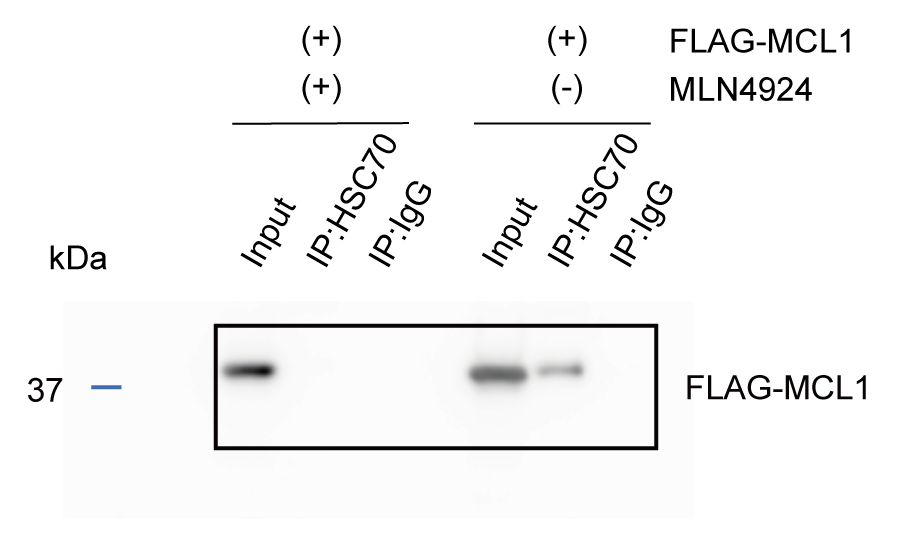

Supplement: Supplementary file 6 — Source data Fig. 5 [file 44319_2025_376_MOESM6_ESM.zip › Fig.5/Fig.5E/FLAG-MCL1/Fig.5E FLAG-MCL1.tif]

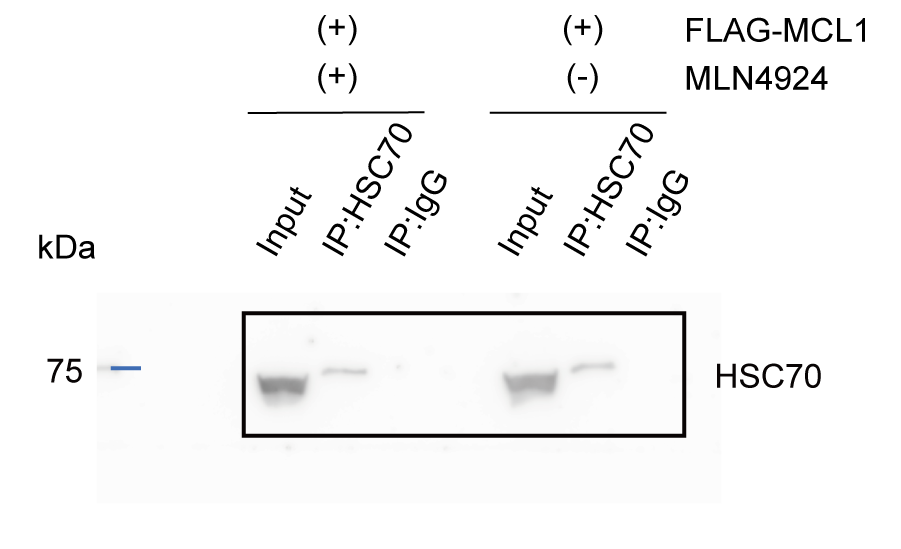

Supplement: Supplementary file 6 — Source data Fig. 5 [file 44319_2025_376_MOESM6_ESM.zip › Fig.5/Fig.5E/HSC70/Fig.5E HSC70.tif]

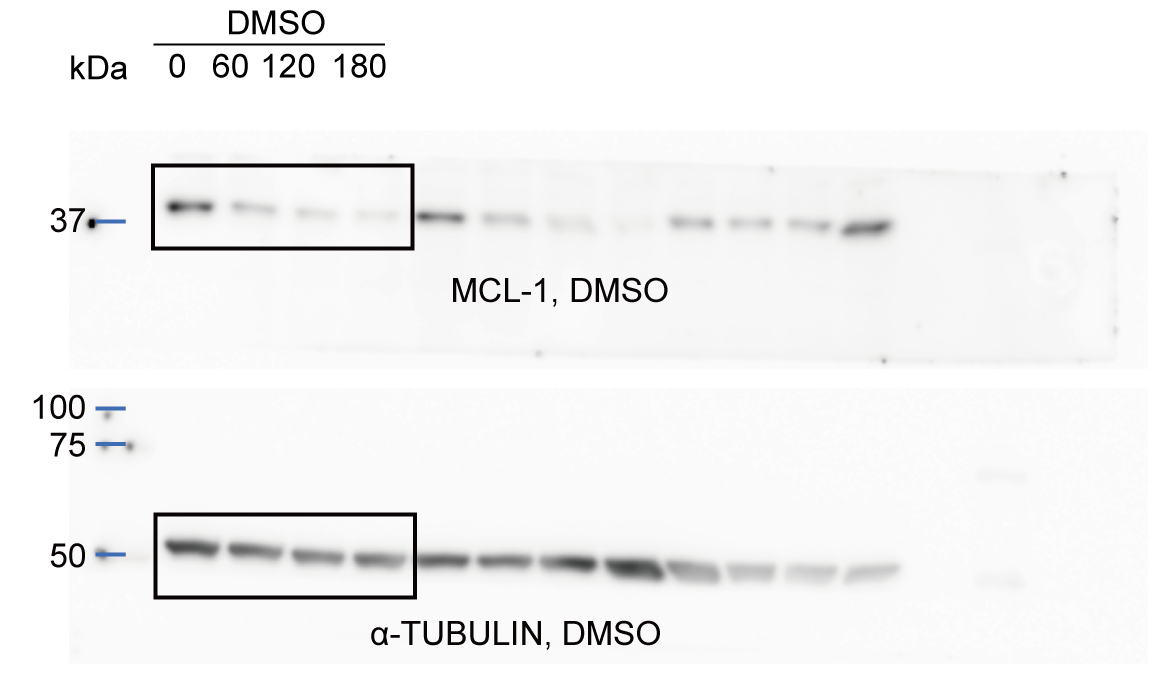

Supplement: Supplementary file 7 — Source data Fig. 6 [file 44319_2025_376_MOESM7_ESM.zip › Fig.6/Fig.6A/DMSO/Fig.6A DMSO MCL1, α-TUBULIN.tif]

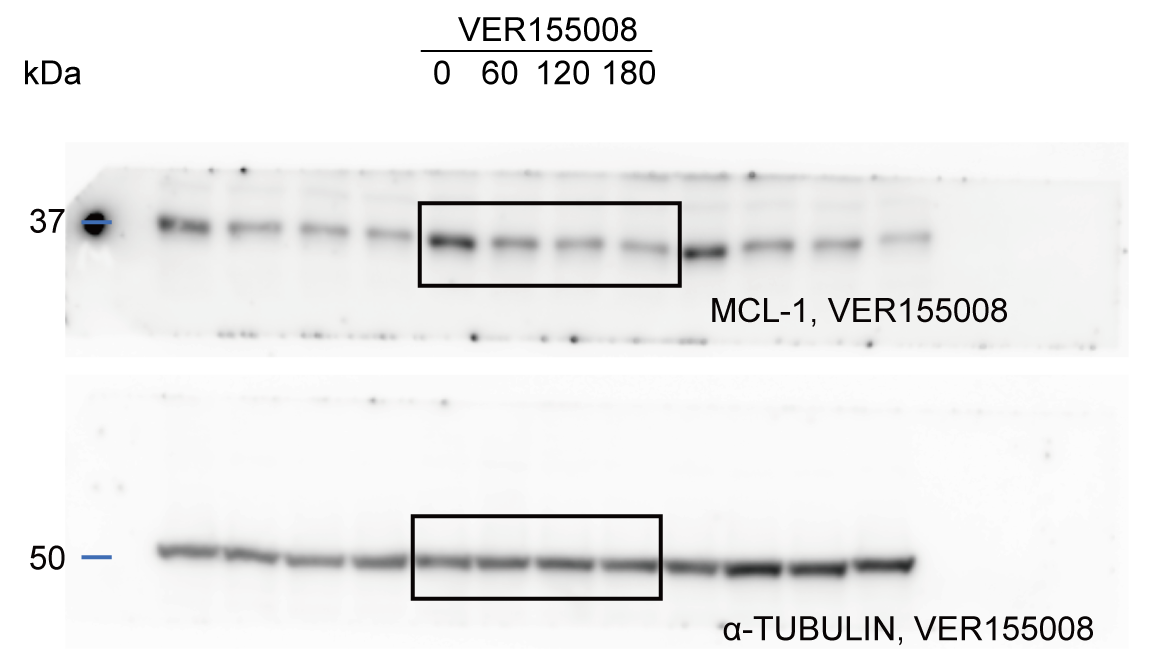

Supplement: Supplementary file 7 — Source data Fig. 6 [file 44319_2025_376_MOESM7_ESM.zip › Fig.6/Fig.6A/VER155008/Fig.6A VER155008 MCL1, α-TUBULIN.tif]

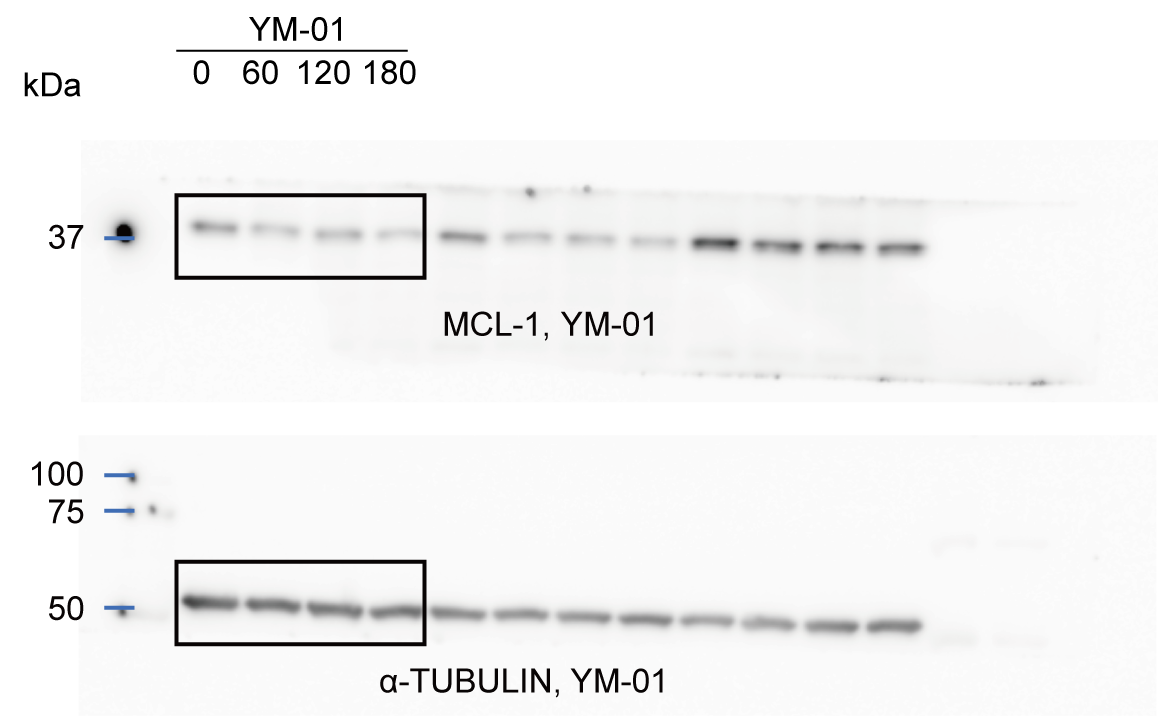

Supplement: Supplementary file 7 — Source data Fig. 6 [file 44319_2025_376_MOESM7_ESM.zip › Fig.6/Fig.6A/YM-01/Fig.6A YM-01 MCL1, α-TUBULIN.tif]

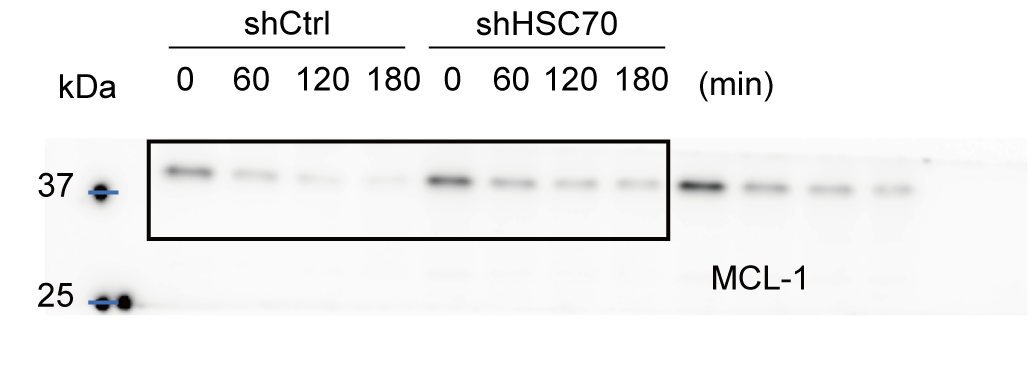

Supplement: Supplementary file 7 — Source data Fig. 6 [file 44319_2025_376_MOESM7_ESM.zip › Fig.6/Fig.6B/MCL1/Fig.6B MCL1.tif]

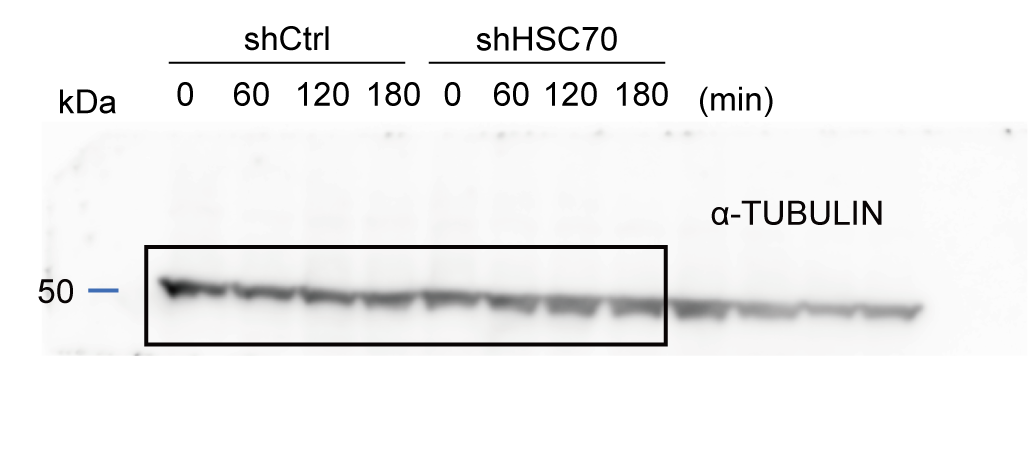

Supplement: Supplementary file 7 — Source data Fig. 6 [file 44319_2025_376_MOESM7_ESM.zip › Fig.6/Fig.6B/α-TUBULIN/Fig.6B α-TUBLIN.tif]

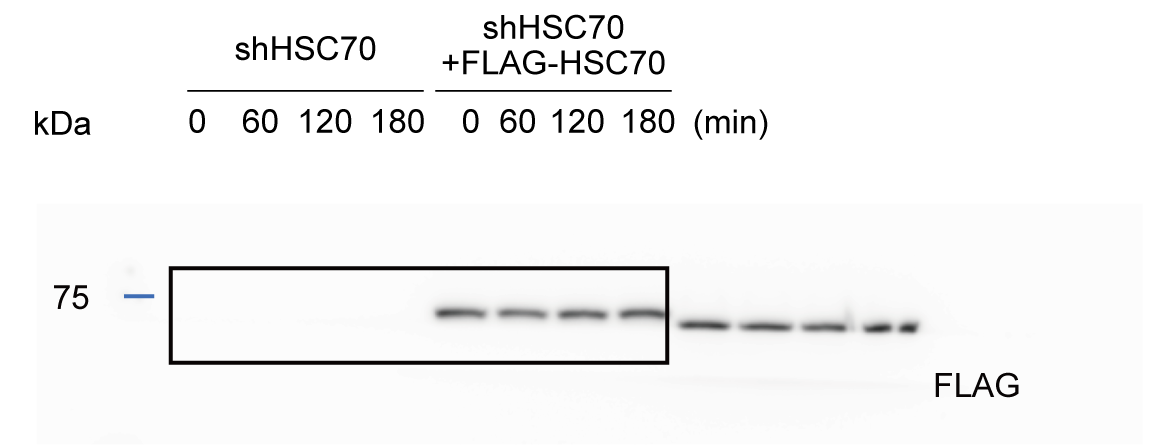

Supplement: Supplementary file 7 — Source data Fig. 6 [file 44319_2025_376_MOESM7_ESM.zip › Fig.6/Fig.6C/FLAG-HSC70/Fig.6C FLAG-HSC70.tif]

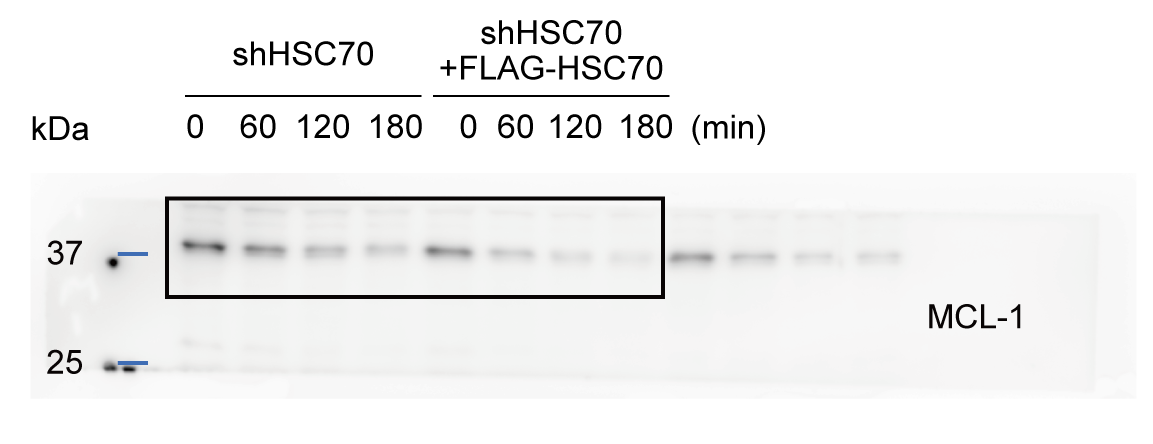

Supplement: Supplementary file 7 — Source data Fig. 6 [file 44319_2025_376_MOESM7_ESM.zip › Fig.6/Fig.6C/MCL1/Fig.6C MCL-1.tif]

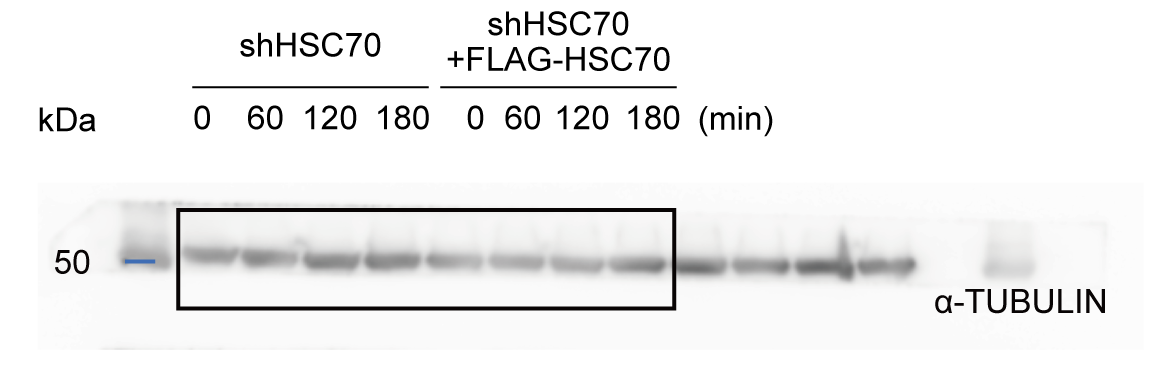

Supplement: Supplementary file 7 — Source data Fig. 6 [file 44319_2025_376_MOESM7_ESM.zip › Fig.6/Fig.6C/α-TUBULIN/Fig.6C α-TUBULIN.tif]

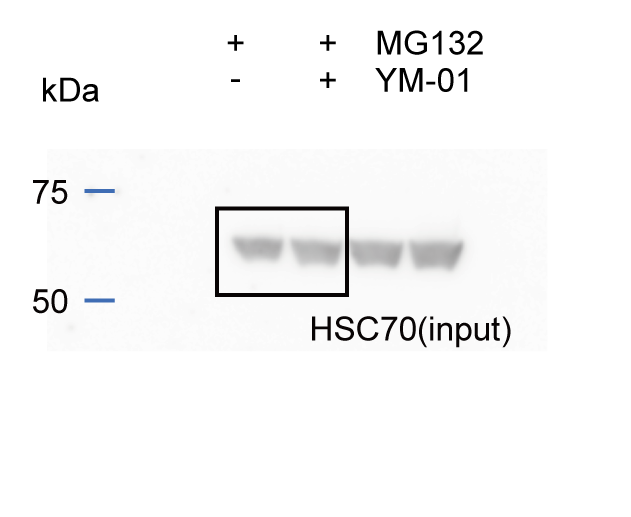

Supplement: Supplementary file 7 — Source data Fig. 6 [file 44319_2025_376_MOESM7_ESM.zip › Fig.6/Fig.6D/HSC70 input/Fig.6D HSC70(input).tif]

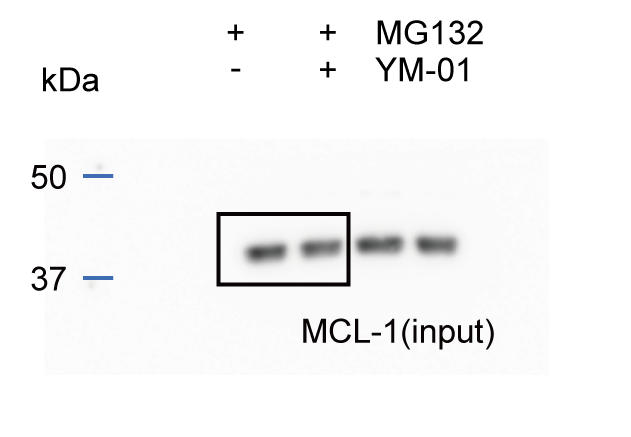

Supplement: Supplementary file 7 — Source data Fig. 6 [file 44319_2025_376_MOESM7_ESM.zip › Fig.6/Fig.6D/MCL1 input/Fig.6D MCL1(input).tif]

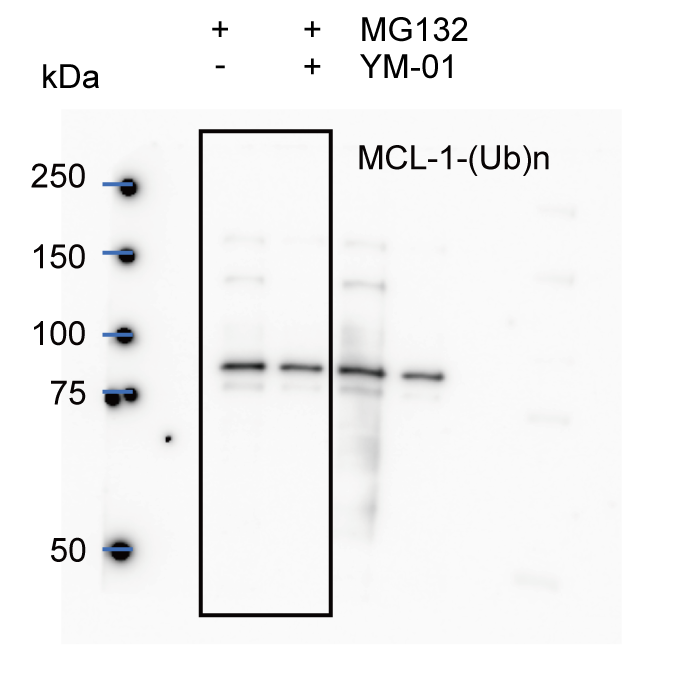

Supplement: Supplementary file 7 — Source data Fig. 6 [file 44319_2025_376_MOESM7_ESM.zip › Fig.6/Fig.6D/MCL1 Pulldown/Fig.6D TUBE2 Pulldown MCL1.tif]

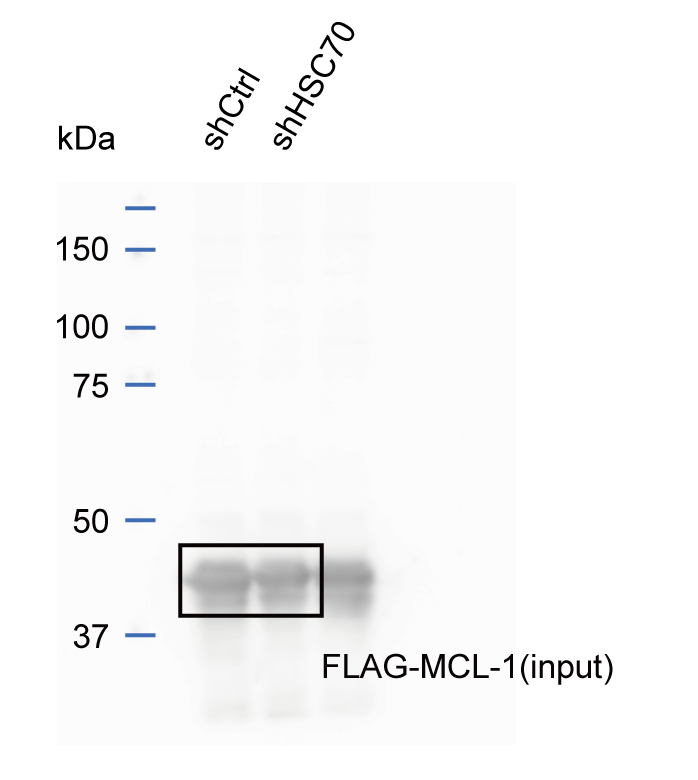

Supplement: Supplementary file 7 — Source data Fig. 6 [file 44319_2025_376_MOESM7_ESM.zip › Fig.6/Fig.6E/FLAG-MCL1 input/Fig.6E MCL1(input).tif]

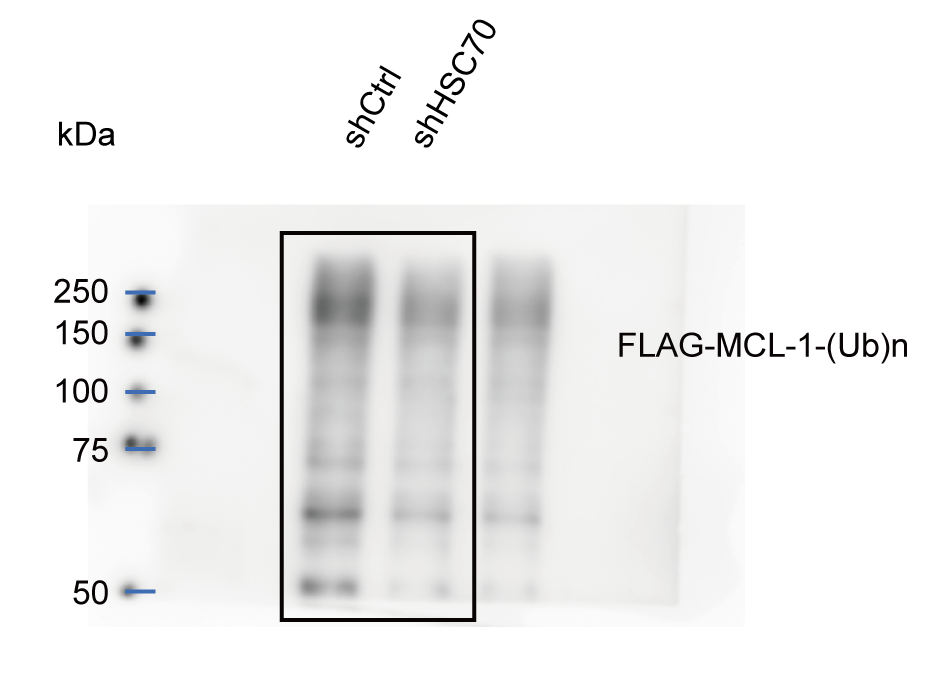

Supplement: Supplementary file 7 — Source data Fig. 6 [file 44319_2025_376_MOESM7_ESM.zip › Fig.6/Fig.6E/FLAG-MCL1 pulldown/Fig.6E TUBE2 Pulldown FLAG-MCL1.tif]

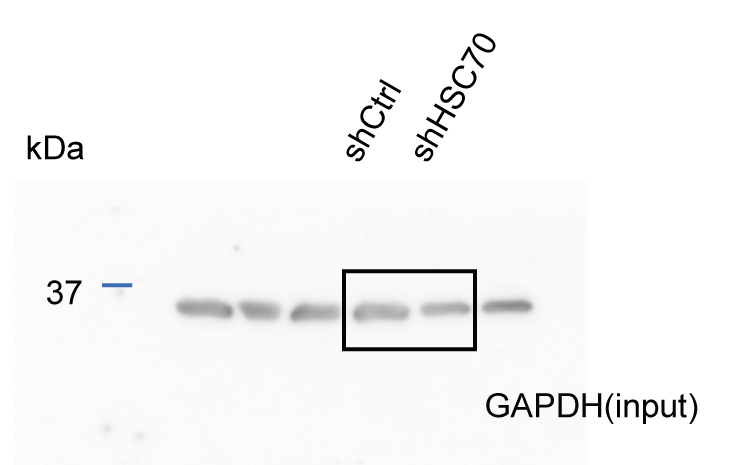

Supplement: Supplementary file 7 — Source data Fig. 6 [file 44319_2025_376_MOESM7_ESM.zip › Fig.6/Fig.6E/GAPDH input/Fig.6E GAPDH(input).tif]

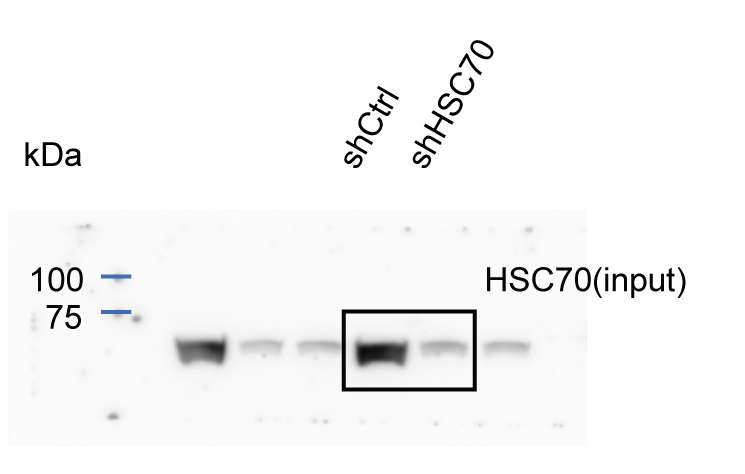

Supplement: Supplementary file 7 — Source data Fig. 6 [file 44319_2025_376_MOESM7_ESM.zip › Fig.6/Fig.6E/HSC70 input/Fig.6E HSC70(input).tif]

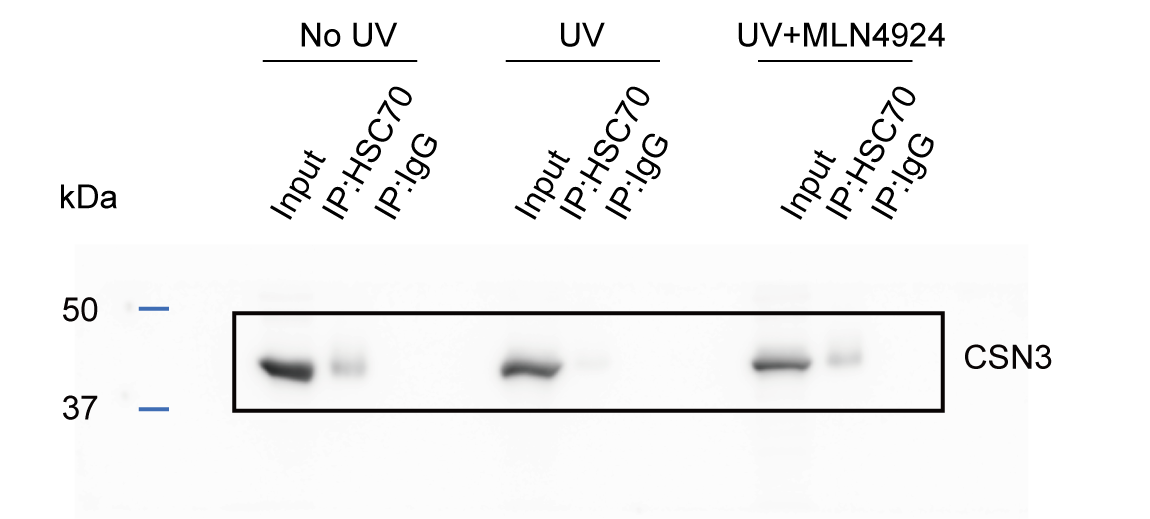

Supplement: Supplementary file 8 — Source data Fig. 7 [file 44319_2025_376_MOESM8_ESM.zip › Fig.7/Fig.7A/CSN3/Fig.7A CSN3.tif]

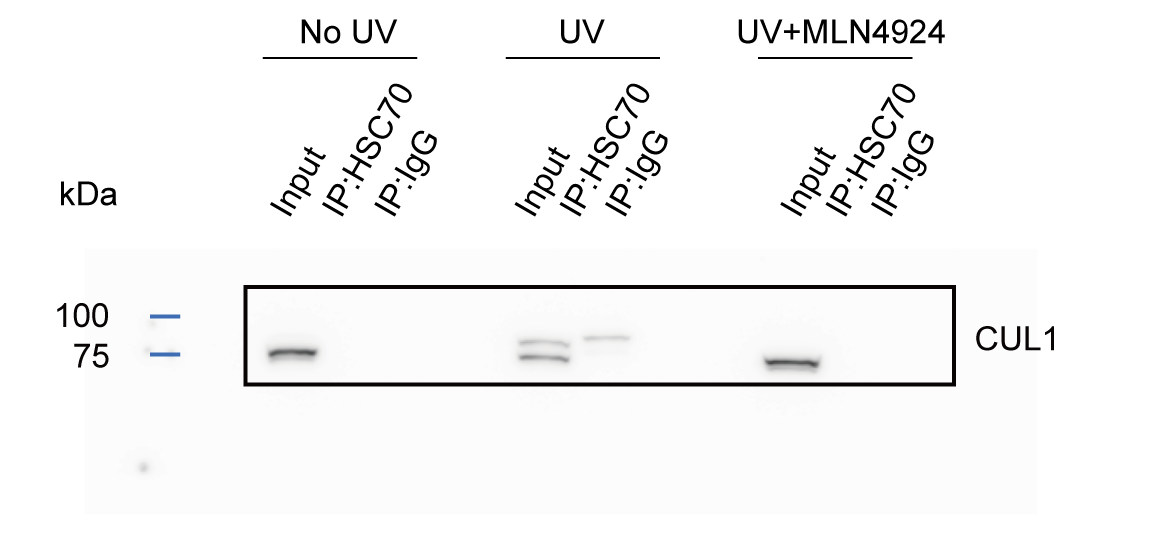

Supplement: Supplementary file 8 — Source data Fig. 7 [file 44319_2025_376_MOESM8_ESM.zip › Fig.7/Fig.7A/CUL1/Fig.7A CUL1.tif]

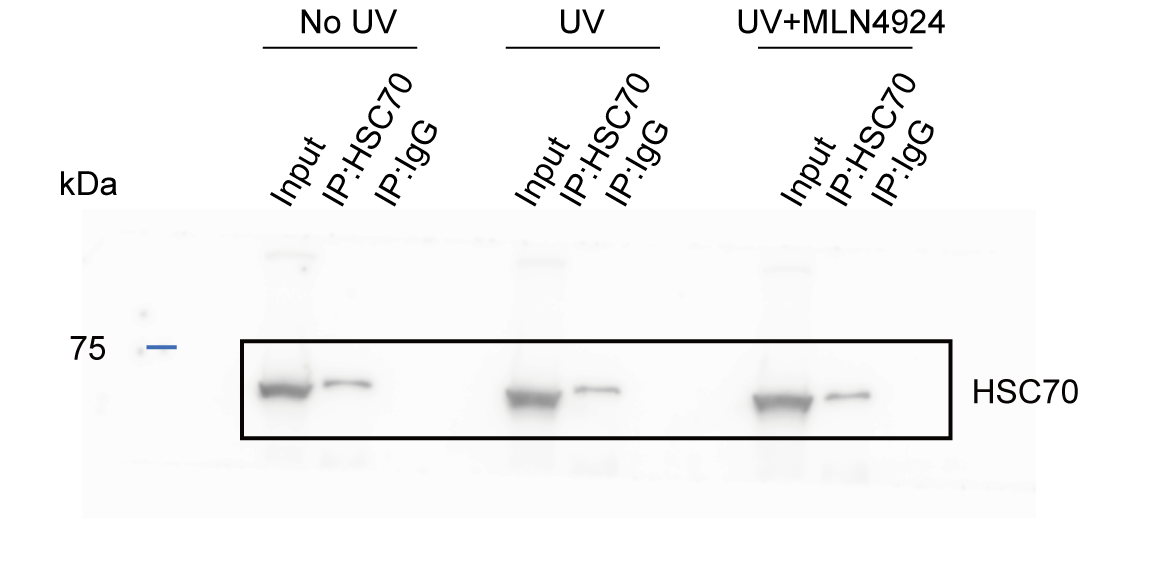

Supplement: Supplementary file 8 — Source data Fig. 7 [file 44319_2025_376_MOESM8_ESM.zip › Fig.7/Fig.7A/HSC70/Fig.7A HSC70.tif]

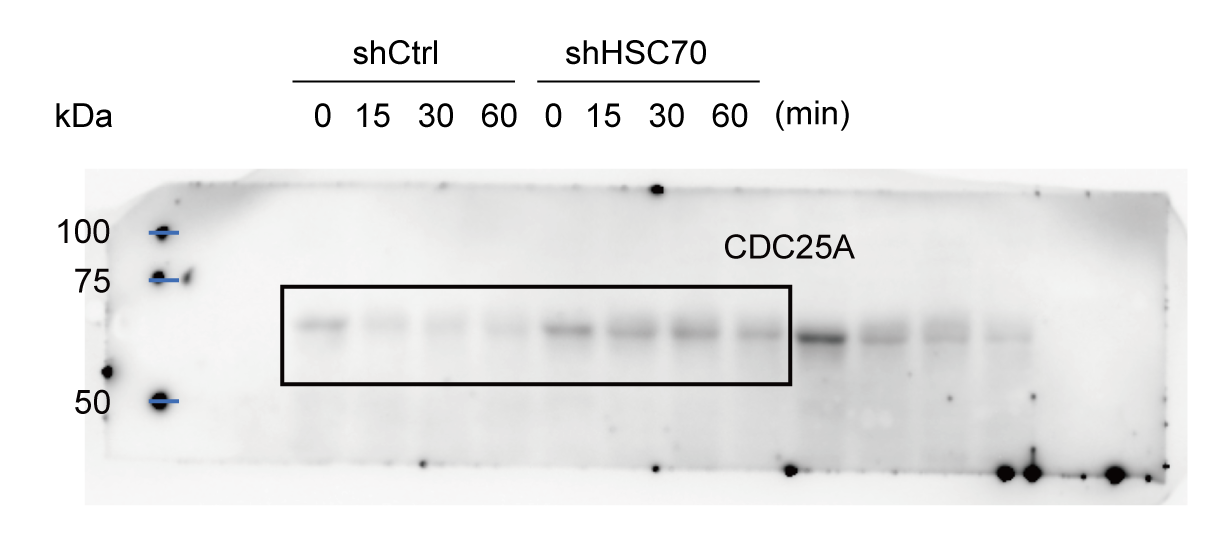

Supplement: Supplementary file 8 — Source data Fig. 7 [file 44319_2025_376_MOESM8_ESM.zip › Fig.7/Fig.7B/CDC25A/Fig.7B CDC25A.tif]

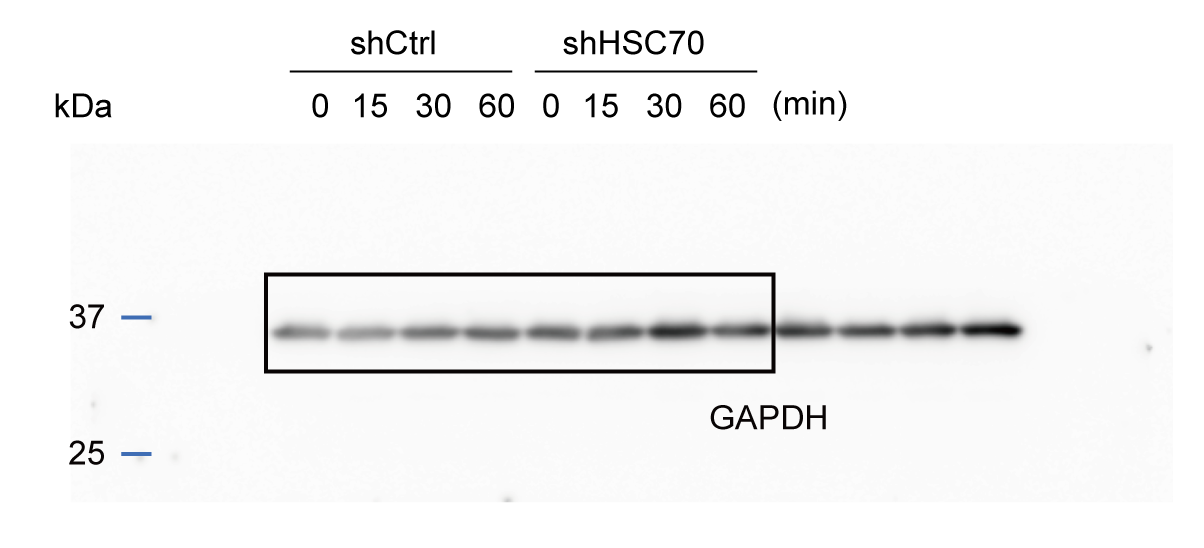

Supplement: Supplementary file 8 — Source data Fig. 7 [file 44319_2025_376_MOESM8_ESM.zip › Fig.7/Fig.7B/GAPDH/Fig.7B GAPDH.tif]

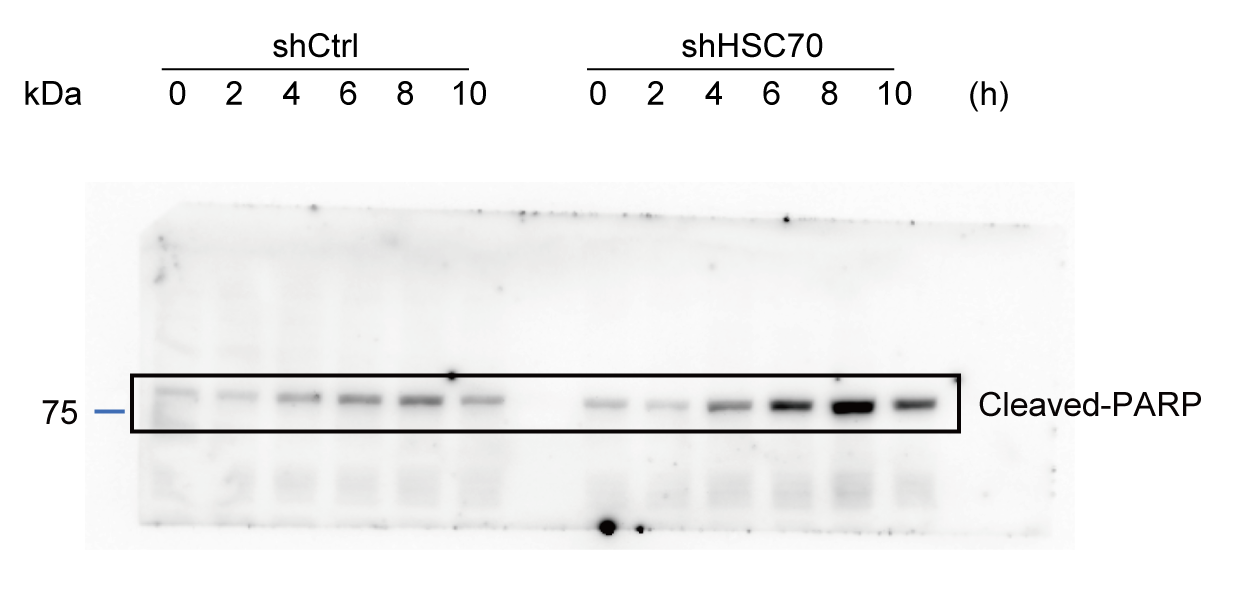

Supplement: Supplementary file 8 — Source data Fig. 7 [file 44319_2025_376_MOESM8_ESM.zip › Fig.7/Fig.7E/Cleaved-PARP/Fig.7E Cleaved-PARP.tif]

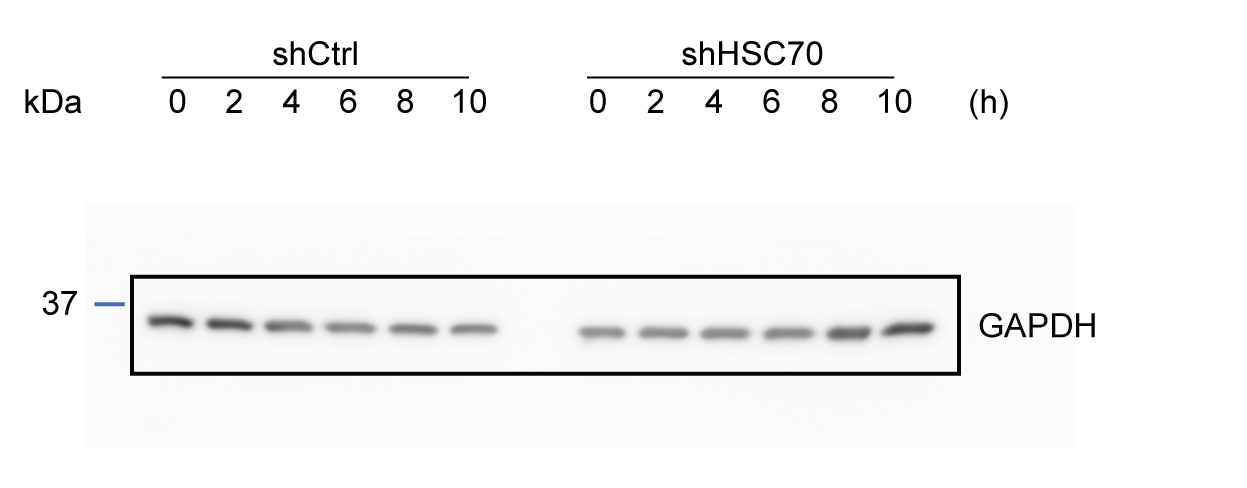

Supplement: Supplementary file 8 — Source data Fig. 7 [file 44319_2025_376_MOESM8_ESM.zip › Fig.7/Fig.7E/GAPDH/Fig.7E GAPDH.tif]
