# Supplementary material for: HSC70 coordinates COP9 signalosome and SCF ubiquitin ligase activity to enable a prompt stress response
Source: EMBO Rep. 2025 Feb 6;26(5):1344–66. doi: 10.1038/s44319-025-00376-x (PMC11894141; doi:10.1038/s44319-025-00376-x)
Supplement: Supplementary file 9 — Expanded View Figures [file 44319_2025_376_MOESM9_ESM.pdf]

Expanded View Figures

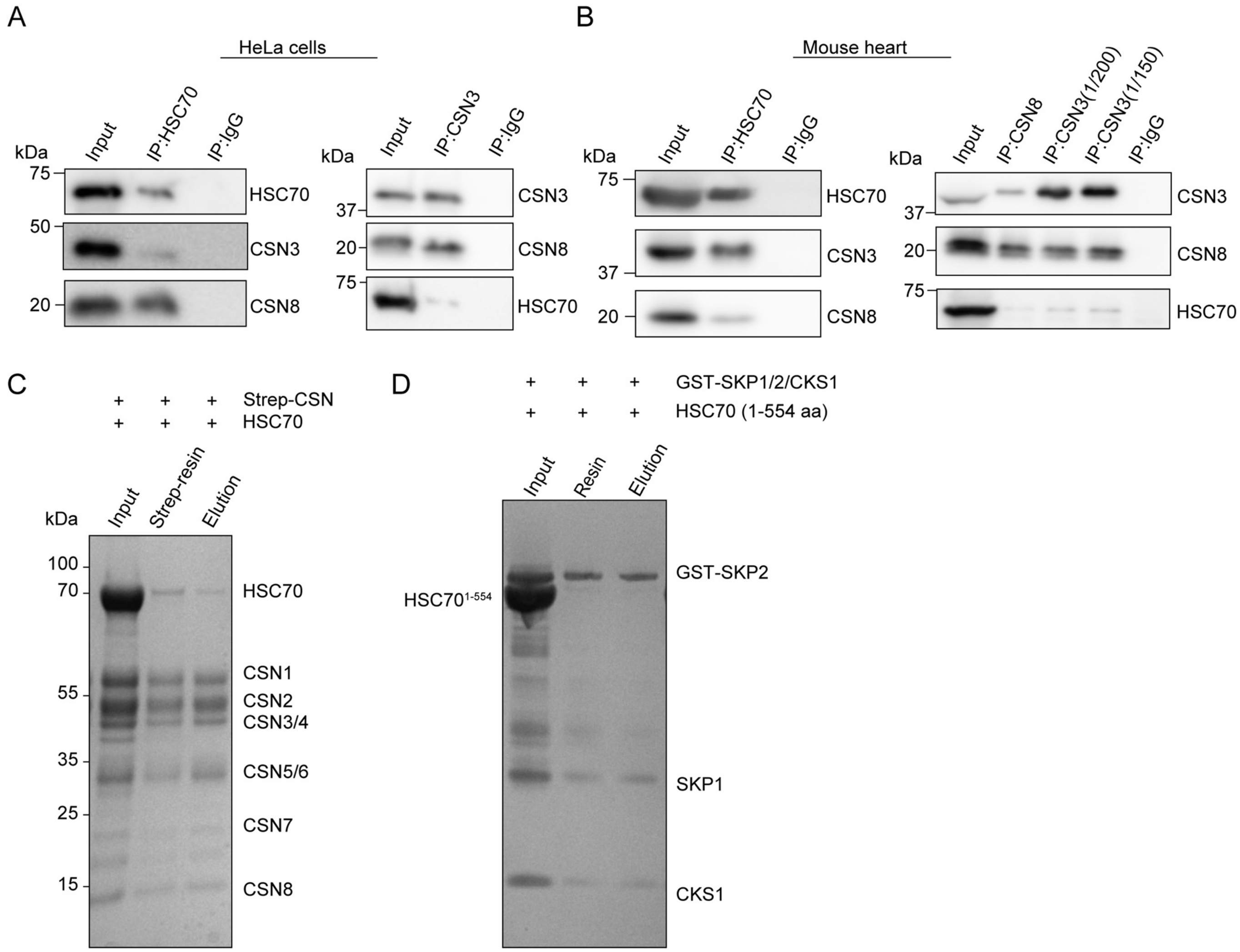

Figure EV1. HSC70 interacts with CSN in vivo and in vitro, but does not interacts with SKP1/2/CKS mini complex.

(**A**) Co-immunoprecipitation in HeLa cells using HSC70 and CSN3 antibody, with rabbit-IgG serving as a negative control. (**B**) Co-immunoprecipitation in mouse heart tissue using HSC70, CSN8, and CSN3 antibody (1/150 or 1/200 dilution), with rabbit-IgG serving as a negative control. (**C**) In vitro pulldown assay. A mixture containing strep-tagged CSN and recombinant HSC70 was subjected to immunoprecipitation with Streptactin Sepharose. The result was visualized by Coomassie brilliant blue staining. (**D**) In vitro pulldown assay. A mixture containing GST-tagged SKP1/2/CKS1 and $HSC70^{1\text{-}554aa}$ was subjected to immunoprecipitation with Glutathione Sepharose 4 Fast Flow. The results were visualized by Coomassie brilliant blue staining.

 © The Author(s)

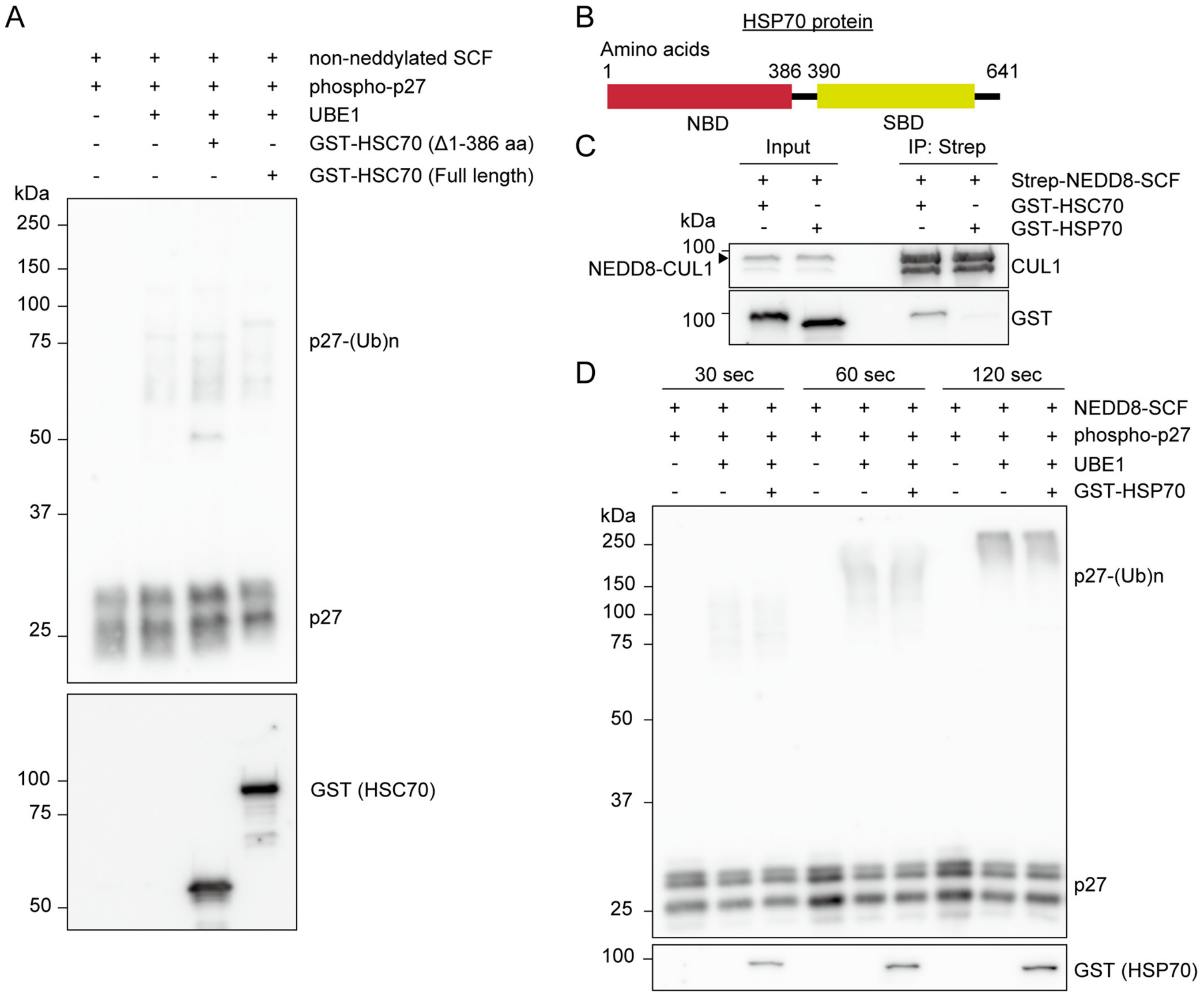

Figure EV2. HSC70 does not enhance the ubiquitination activity of non-neddylated SCF, and HSP70 does not enhance NEDD8-SCF ubiquitination activity.

(**A**) Non-neddylated SCF mediated in vitro ubiquitination of p-p27 assayed in the absence or presence of indicated recombinant proteins. Unmodified and polyubiquitinated p-p27 were detected by immunoblotting with anti-p27 antibody. (**B**) Schematic diagram of HSP70 protein structure. (**C**) In vitro pulldown assay. A mixture containing GST-tagged HSC70 or HSP70, and Strep-tagged NEDD8-SCF were subjected to immunoprecipitation with Streptactin Sepharose. (**D**) NEDD8-SCF mediated in vitro ubiquitination of p-p27 assayed in the absence or presence of GST-HSP70.

© The Author(s)

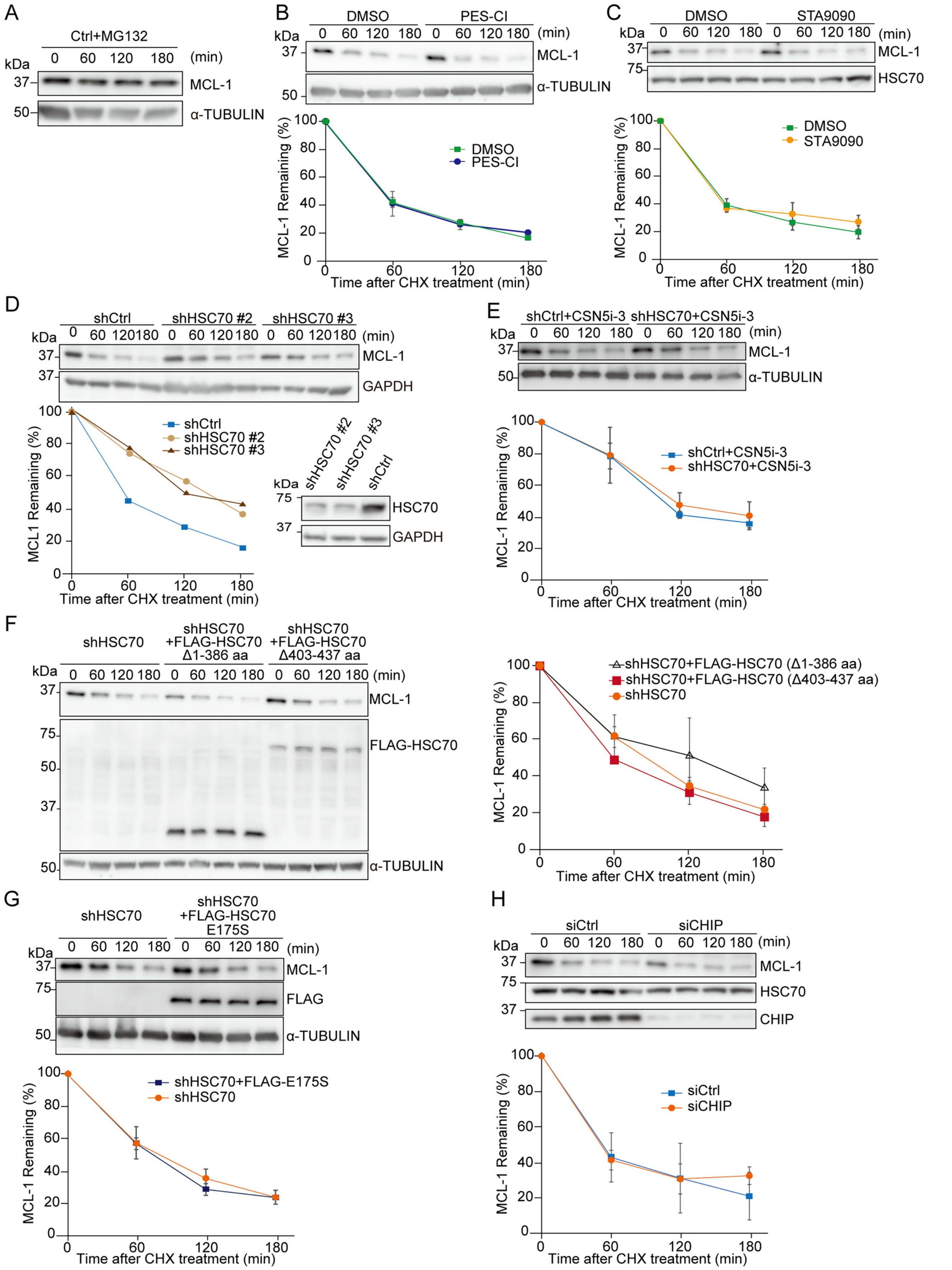
A
Ctrl+MG132
kDa 0 60 120 180 (min)
37 MCL-1
50 α-TUBULIN
B
DMSO PES-Cl
kDa 0 60 120 180 0 60 120 180 (min)
37 MCL-1
50 α-TUBULIN
DMSO
PES-Cl
MCL-1 Remaining (%)
Time after CHX treatment (min)
C
DMSO STA9090
kDa 0 60 120 180 0 60 120 180 (min)
37 MCL-1
75 HSC70
DMSO
STA9090
MCL-1 Remaining (%)
Time after CHX treatment (min)
D
shCtrl shHSC70 #2 shHSC70 #3
kDa 0 60 120180 0 60 120 180 0 60 120180 (min)
37 MCL-1
37 GAPDH
shCtrl
shHSC70 #2
shHSC70 #3
MCL1 Remaining (%)
Time after CHX treatment (min)
kDa shHSC70 #2 shHSC70 #3 shCtrl
75 HSC70
37 GAPDH
E
shCtrl+CSN5i-3 shHSC70+CSN5i-3
kDa 0 60 120 180 0 60 120 180 (min)
37 MCL-1
50 α-TUBULIN
shCtrl+CSN5i-3
shHSC70+CSN5i-3
MCL-1 Remaining (%)
Time after CHX treatment (min)
F
shHSC70
shHSC70 +FLAG-HSC70 Δ1-386 aa
shHSC70 +FLAG-HSC70 Δ403-437 aa
kDa 0 60 120 180 0 60 120 180 0 60 120 180 (min)
37 MCL-1
75 50 37 FLAG-HSC70
50 α-TUBULIN
shHSC70+FLAG-HSC70 (Δ1-386 aa)
shHSC70+FLAG-HSC70 (Δ403-437 aa)
shHSC70
MCL-1 Remaining (%)
Time after CHX treatment (min)
G
shHSC70
shHSC70 +FLAG-HSC70 E175S
kDa 0 60 120 180 0 60 120 180 (min)
37 MCL-1
75 FLAG
50 α-TUBULIN
shHSC70+FLAG-E175S
shHSC70
MCL-1 Remaining (%)
Time after CHX treatment (min)
H
siCtrl siCHIP
kDa 0 60 120 180 0 60 120 180 (min)
37 MCL-1
75 HSC70
37 CHIP
siCtrl
siCHIP
MCL-1 Remaining (%)
Time after CHX treatment (min)

Figure EV3. Cycloheximide (CHX) chase assay in various conditions.

(**A**) CHX chase assay of endogenous MCL-1 in the presence of proteasome inhibitor MG132. (**B**) CHX chase assay of endogenous MCL1 with pharmacological inhibition with PES-Cl. The graphs show the mean ± s.e.m. ($n = 3$, respectively; Welch's t test). No significant difference was observed at each time point. (**C**) CHX chase assay of endogenous MCL1 with pharmacological inhibition with STA9090. The graphs show the mean ± s.e.m. ($n = 3$, respectively; Welch's t test). No significant difference was observed at each time point. (**D**) CHX chase assay of endogenous MCL-1 in other *HSC70* knockdown cell lines (shHSC70 clones #2 and 3). The lower right panel shows the extent of *HSC70* knockdown in these cell lines by immunoblotting. (**E**) CHX chase assay of endogenous MCL1 in *HSC70* knockdown cells with pharmacological inhibition with CSN5i-3. The graphs show the mean ± s.e.m. ($n = 3$, respectively; Welch's t test). No significant difference was observed at each time point. (**F**) CHX chase assay of endogenous MCL-1 with or without transient FLAG-HSC70 mutant expression. The graphs show the mean ± s.e.m. (Δ1–386 aa or Δ403–437 aa; $n = 3$, respectively; Welch's t test). No significant difference was observed at each time point. (**G**) CHX chase assay of endogenous MCL-1 with or without transient FLAG-HSC70 E175S mutant expression. The graphs show the mean ± s.e.m. ($n = 3$, respectively; Welch's t-test). No significant difference was observed at each time point. (**H**) CHX chase assay of endogenous MCL1 with or without *CHIP* knockdown. The graphs show the mean ± s.e.m. ($n = 3$, respectively; Welch's t test). No significant difference was observed at each time point. (**B–H**) Each protein level was densitometrically quantified (normalized to 0 min) and shown in the graph below.

© The Author(s)

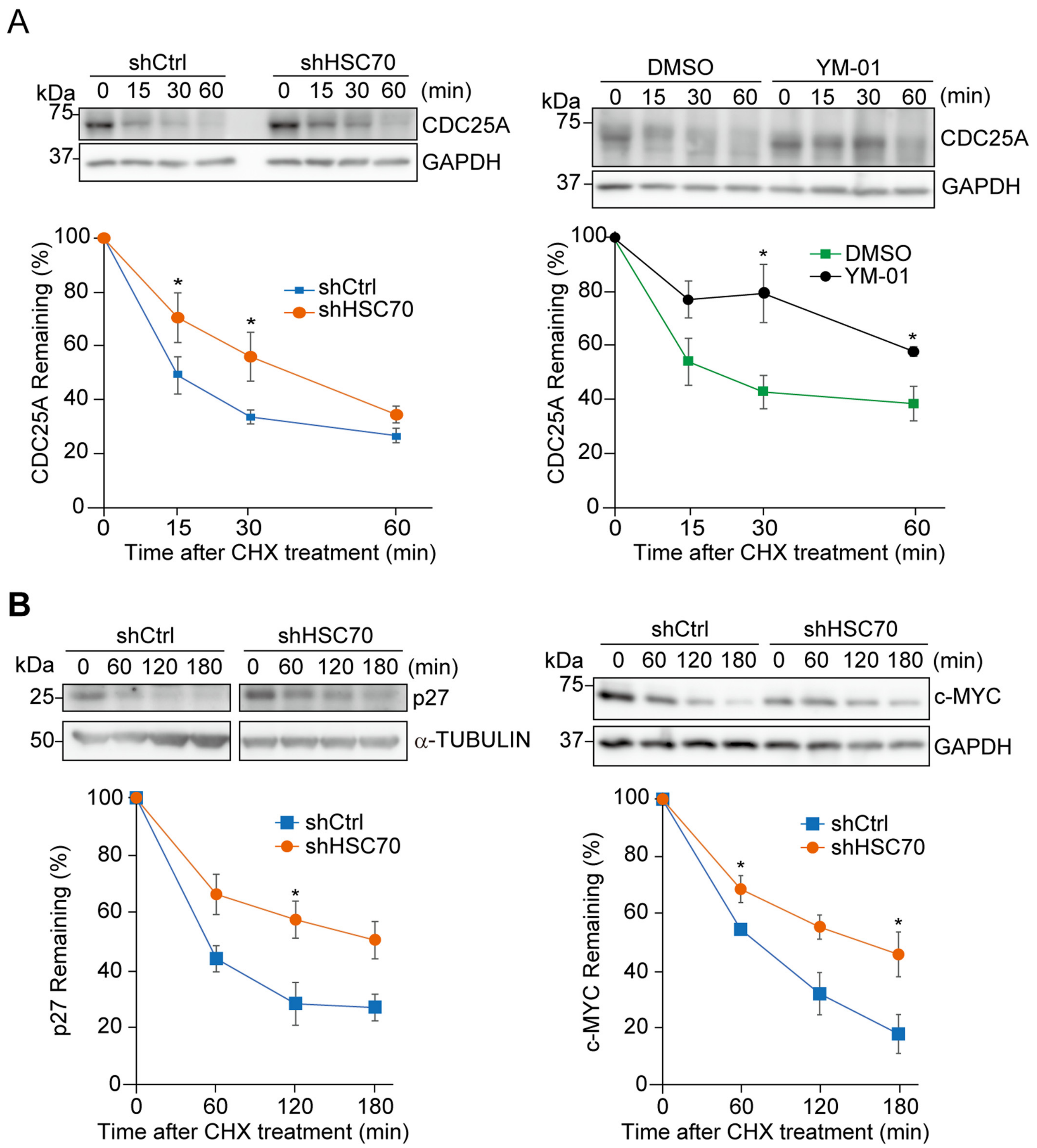

Figure EV4. Pharmacological or genetic inhibition of HSC70 prolonged the half-life of other SCF substrates.

(**A**) CHX chase assay of endogenous CDC25A with or without *HSC70* knockdown, or pharmacological inhibition with YM-01. The bottom graphs show the mean ± s.e.m. $^*P < 0.05$ ($n = 3$ independent experiments; Welch's t test). Compared with shCtrl cells or DMSO treatment, a significant difference was observed at 15 min ($P = 0.025$) and 30 min ($P = 0.045$) in shHSC70 cells and at 30 min ($P = 0.042$) and 60 min ($P = 0.011$) in YM-01 treated cells. (**B**) CHX chase assay of endogenous p27 or c-MYC with or without *HSC70* knockdown. The bottom graphs show the mean ± s.e.m. $^*P < 0.05$ ($n = 3$ independent experiments, respectively; Welch's t test). For p27 protein, a significant difference was observed at 120 min ($P = 0.032$). For c-MYC protein, significant difference was observed at 60 min ($P = 0.033$) and 180 min ($P = 0.045$). (**A**, **B**) Each protein level was densitometrically quantified (normalized to 0 min) and shown in the graph below.

© The Author(s)

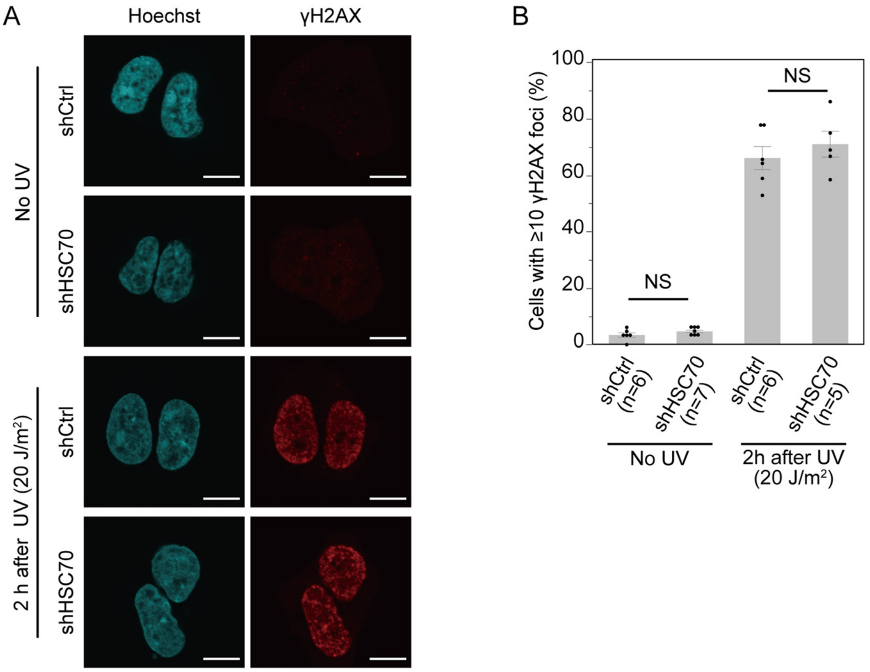

Figure EV5. Ultraviolet-induced DNA damage in HSC70 knockdown cells.

(**A**) Immunostaining of control or HSC70 knockdown HEK293T cells with or without low-dose UV irradiation (20 J/m^2) using γH2AX antibody and Hoechst 33342. Scale bars, 10 µm. (**B**) Quantification of cells with γH2AX positive foci with or without UV irradiation ($n = 6$ independent experiments for shCtrl cells with or without UV, respectively; $n = 7$ or 5 independent experiments for shHSC70 cells with or without UV, respectively; Welch's t test). The graphs show the mean ± s.e.m. NS not significant.
